# Mineral-Based Advanced Oxidation Processes for Enhancing the Removal of Antibiotic Resistance Genes from Domestic Wastewater

**DOI:** 10.1021/acsestwater.4c01213

**Published:** 2025-04-29

**Authors:** Panagiota Adamou, James Entwistle, David W. Graham, Anke Neumann

**Affiliations:** † School of Engineering, Newcastle University, Newcastle upon Tyne NE1 7RU, U.K.; ‡ PSI Center for Nuclear Engineering and Sciences, 5232 Villigen PSI, Switzerland

**Keywords:** clay mineral, antimicrobial resistance, advanced oxidation process, iron, tertiary treatment, wastewater treatment plant effluent

## Abstract

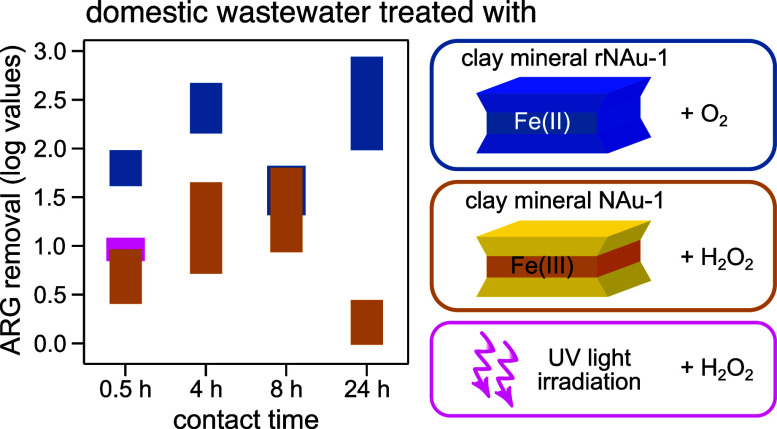

Wastewater treatment plants (WWTPs) release antibiotic-resistant bacteria (ARB) and antibiotic-resistant genes (ARGs) into the environment. Advanced oxidation processes (AOPs) can remove ARB and ARGs, but they often require impractically high chemical or energy use. Here, we explore a low-energy AOP that uses Fe-bearing clay mineral (NAu-1) either combined with H_2_O_2_ (H_2_O_2_/NAu-1) or as prereduced structural Fe (rNAu-1) to degrade selected ARGs (i.e., *tet*M, *tet*Q, and *bla*_OXA-10_), *int*1 (a mobile genetic element), and the 16S rRNA gene in postsecondary WWTP effluents. Addition of H_2_O_2_/NAu-1 significantly increased *tet*M and *int*1 removals relative to UV irradiation and H_2_O_2_/UV (*p* ≤ 0.02). Removals increased with greater H_2_O_2_ doses and contact times, reaching maximum values of 1.2 and 2.3 log units at H_2_O_2_ doses of 0.26 and 10 mM and contact times of 4 and 8 h, respectively. Bacterial regrowth after 24 h of contact was probably due to H_2_O_2_ depletion. However, the addition of rNAu-1 achieved the highest removals, up to 2.9 log units after 0.5 h, and suppressed bacterial regrowth over 24 h. Similar removals were observed with rNAu-1 under oxic and anoxic conditions. Results show that mineral-based AOPs offer the potential for elevated ARG removal and lower chemical and energy demands in tertiary wastewater treatment.

## Abstract 2

This work reports rapid and effective bacteria and antimicrobial resistance gene removal using iron-bearing clay mineral. This advanced oxidation process holds potential for the sustainable tertiary treatment of wastewater.

## Introduction

Antimicrobial resistance (AMR) is a global concern,^1^ and one of the most common pathways of antibiotic-resistant bacteria (ARB) and genes (ARGs) spread is human wastewater releases to the environment.^2,3^ While modern wastewater treatment plants (WWTPs) effectively remove most pathogens and major nutrients, they do not fully eliminate ARB and ARGs from their effluents,^4^ presenting an important source of AMR to the environment.^5^ Secondary treatment technologies, such as activated sludge, anaerobic–anoxic–aerobic (A_2_O) systems, biofilters, and sequential batch reactors (SBRs), display differing but often acceptable ARB/ARG removal rates,^6−8^ but under particularly sensitive receiving water conditions, tertiary/quaternary treatment can further reduce AMR release in WWTP effluents.^5^ Disinfection technologies, such as chlorination, ozonation, and UV irradiation, have been shown to reduce ARB and ARG levels in WWTP effluents,^4^ yet elevated costs and energy and chemical demands^9,10^ usually make them uneconomical for routine use.

Among tertiary treatment options, advanced oxidation processes (AOPs) have the greatest potential for reducing AMR levels in pretreated wastewater.^2,11^ AOPs, e.g., H_2_O_2_/UV, homogeneous (Fe(II), Fe(III)/H_2_O_2_), or heterogeneous (photo)catalysis (UV/TiO_2_), rely on the formation of reactive oxygen species (ROS), such as hydroxyl radicals (•OH), with highly positive reduction potentials^12^ and nonselective reactivity toward a wide range of organic and inorganic compounds.^11,13^ Although AOPs are being considered for removing ARB and ARGs from wastewater,^11^ application of UV and/or ozonation has high operating and energy costs and requires process optimization,^2^ suggesting more sustainable technologies are needed that use less energy and/or chemicals to promote ROS formation.

Here, we build upon previous work that has shown natural minerals, such as Fe-bearing clay minerals, can efficiently convert H_2_O_2_ to hydroxyl radicals in a Fenton-like process,^14^ accelerating the degradation of target chemicals. In contrast to traditional Fenton reactions,^15^ mineral-based AOPs are effective at circum-neutral pH conditions and do not produce waste iron sludge,^14,16^ enhancing their potential for treating organic contaminants.^17,18^ Moreover, clay mineral Fe that is reduced to ferrous Fe (Fe(II)) can, during its reaction with oxygen, also produce a series of ROS, including superoxide and •OH,^19,20^ analogous to what has long been known for dissolved Fe(II).^21^ Oxygenation of clay mineral Fe(II) does not require chemical or energy input, making this mineral-based AOP potentially more sustainable for tertiary wastewater treatment. Despite this potential, only few studies to date have addressed organic contaminant degradation^20,22,23^ or explored the antibacterial properties of natural^24−26^ and reduced (Fe(II)-containing) clay minerals.^27^ Neither H_2_O_2_ activation by native (i.e., Fe(III)-containing) clay minerals nor oxygenation of Fe(II)-containing clay minerals has been studied for removing ARB and ARGs from authentic secondary effluents from WWTPs.

In this proof-of-concept study, we assessed the potential of mineral-based AOPs for the removal of ARGs from authentic wastewater. We used an Fe-rich clay mineral, nontronite NAu-1 (∼20 wt % Fe), as a natural mineral catalyst for H_2_O_2_ activation and, after reduction of its native structural Fe(III) to Fe(II), to produce reactive oxidizing species upon Fe(II) oxygenation without the need to add H_2_O_2_. We evaluated the ability of NAu-1 to reduce selected “model” ARGs associated with the native bacterial communities in secondary effluents across a range of H_2_O_2_ concentrations and contact times and compared our results with the efficiencies of conventional UV and H_2_O_2_/UV technologies.

## Materials and Methods

### Wastewater Samples

Grab samples of domestic wastewater from the secondary clarifier effluent at an activated sludge WWTP in NE England were collected in sterile polypropylene containers (Fisher Scientific, UK), stored at 4 °C, and analyzed/used within 24 h. A portable multimeter (HQ40D; Hach Lange, UK) was used to measure pH, dissolved oxygen (DO), and conductivity (Table S1) on site. Chemical oxygen demand (COD) and total suspended solids (TSS) were analyzed in triplicate using the LCK 314 COD cuvette test (Hach Lange, UK; range: 15–150 mg/L) and according to the standard method,^28^ respectively.

### ARG Removal Experiments

Batch experiments were set up using 250 mL of secondary clarifier effluent (“feedwater”) in 500 mL borosilicate glass beakers (VWR, UK) (“reactors”) and carried out in triplicate at room temperature (22 ± 2 °C) and under constant stirring. Three types of experiments were performed, and the removal of selected native ARGs associated with the bacterial communities in secondary wastewater effluents was assessed.

#### Benchmarking Experiments

Three sets of initial experiments were performed to benchmark ARG removal using UV, H_2_O_2_/UV spectroscopy, and H_2_O_2_/NAu-1. As the main purpose of experiments here was to demonstrate the clay-based approach could work in principle, optimization was not performed in terms of irradiance, contact time, concentrations, and energy use. Experimental conditions were chosen to be consistent with previous work^16,29^ and the experience of Thames Water, the project’s industrial partner.

In set 1 (UV), a germicidal UV lamp (15 W, 254 nm wavelength; model SC8D, Eurodyne, UK) was used to provide a fixed UV irradiance (320 μW/cm^2^, measured with a UVP radiometer (VWR, UK)).^9^ UV doses of 96, 288, and 576 mJ/cm^2^ were applied by varying exposure time (5, 15, and 30 min, respectively)^9^ and fall within and cover the range of doses commonly used in water treatment.^11^ In set 2 (H_2_O_2_/UV), hydrogen peroxide (30% w/v; Fisher Scientific, UK) was added to yield a 20 mM initial concentration as used previously,^29,30^ and UV irradiation was applied as in set 1. To terminate the reaction, aliquots of 1.0 mL were withdrawn, and 20 μL of 2300 units/mg bovine liver catalase (Sigma-Aldrich, UK) were added to eliminate residual H_2_O_2_.^32^ This catalase concentration (0.1 g/L) does not affect bacterial viability.^32^

In set 3, the same initial H_2_O_2_ concentration (20 mM) was used in combination with Fe-rich clay mineral NAu-1 (1 g/L) and reacted with feedwater for 8 h in the dark.^16^ NAu-1 (M_1.05_^+^[Si_6.98_Al_1.02_][Al_0.29_Fe_3.68_Mg_0.04_]O_20_OH_4_,^33^ measured Fe content of 19.8 wt %^34^) was purchased from the Source Clays Repository of The Clay Minerals Society (www.clays.org), dried, crushed in a ball mill, size-fractionated to ≤2 μm particles, freeze-dried,^34^ and autoclaved prior to adding to the reactors. Addition of bovine liver catalase terminated the reaction. Additions of NAu-1 only and H_2_O_2_ only to feedwater were run as controls under the same experimental conditions.

#### ARG Removal with H_2_O_2_/NAu-1

The effect of reaction conditions during H_2_O_2_/NAu-1 treatment on ARG removal was next evaluated, but at a lower, more practically relevant NAu-1 concentration of 0.5 g/L. In the first experiments, NAu-1 dose and contact time (8 h) were constant, and different H_2_O_2_ concentrations of 0.1, 0.265, and 10 mM were applied. These doses are within the range used in the full-scale application of H_2_O_2_/UV for removing ARGs in wastewater^35^ or used in previous laboratory ARG removal studies.^29^ In the second experiments, H_2_O_2_ (0.265 mM) and NAu-1 concentrations were kept constant and reacted with feedwater for 30 min, 4 h, and 24 h. The H_2_O_2_ dose was chosen based on the first experiments. Control reactors included addition of NAu-1 only, H_2_O_2_ only, and no additions, i.e., feedwater stirred at room temperature (22 ± 2 °C).

#### ARG Removal during Oxygenation of Reduced Clay Mineral (rNAu-1)

Reactors were assembled inside an anoxic glovebox (100% N_2_, O_2_ < 2 ppm, GS Glovebox Systemtechnik GmbH, Germany), and feedwater was deoxygenated by bubbling with N_2_ for 1 h prior to transfer into the glovebox. NAu-1 was subjected to chemical Fe reduction by an adapted citrate-bicarbonate-dithionite method^34^ and added to the reactors at an initial concentration of 0.5 g/L. The clay mineral Fe reduction extent (Fe(II)/Fe(total)) of this reduced NAu-1 (rNAu-1) was determined after HF digestion using a modified 1,10-phenanthroline method.^36^ A subset of reactors containing rNAu-1 was removed from the glovebox and placed in a dark room with ambient oxygen levels (∼21%, “oxic”), and another subset of reactors remained in the glovebox (“anoxic”). Controls without rNAu-1 added (“no treatment”) were assessed under both oxic and anoxic conditions for the same contact times of 30 min and 4, 8, and 24 h.

### Analytical Procedures

Except for the benchmarking work, the pH was measured (3010 pH meter; Jenway, UK) at the end of each experiment, and 50 mL of suspension was withdrawn from the reactor and centrifuged (4000 rpm, 10 min). The supernatant was filtered (poly(ether sulfone), 0.22 μm; VWR, UK) and divided into two aliquots. One aliquot was mixed with ethanol (1:1) to quench any remaining radicals, stored at 4 °C, and residual H_2_O_2_ concentration was quantified using an adapted version of the titanium(IV) oxysulfate colorimetric method.^37^ The other aliquot was acidified with HCl, stored at 4 °C, and analyzed for Fe(II) and total Fe using the 1,10-phenanthroline method.^38^

For selected samples, additional parameters were determined. Suspension samples were analyzed for COD and TSS, after centrifugation for total phosphorus (TP) using the phosphate Ortho/Total cuvette test (HACH, UK), and after additional filtration (poly(ether sulfone) 0.45 μm; VWR, UK) for total organic carbon (TOC) using a TOC/TN_b_ Analyzer (vario TOC cube; Elementar UK Ltd.). Solid samples from reactors containing rNAu-1 were retrieved by filtration of 20 mL (cellulose ester, 0.2 μm; Millipore, UK) and immediately sealed between two pieces of Kapton tape to prevent oxidation during transfer to the Mössbauer spectrometer. Details of the Mössbauer analysis are provided in the Supporting Information.

Remaining reactor volumes (200 mL) were filtered (poly(ether sulfone), 0.22 μm; Millipore, UK) to capture bacterial cells. DNA captured on the membrane was extracted using the Fast DNA Spin Kit for Soil (MP Biomedicals, USA), its quality assessed spectrophotometrically (NanoDrop 2000C, NanoDrop Technologies, USA), and quantified using the Qubit dsDNA HS Assay Kits (Fisher Scientific, UK) in conjunction with the Qubit 2.0 Fluorometer (Thermo Fisher Scientific, UK). Extracted DNA samples were stored at −20 °C until further use. Gene quantification was performed using qPCR for the 16S rRNA, *int*1, *bla*_OXA-10_, *tet*M, and *tet*Q genes using specific primers (see Table S2), SsoFast EvaGreen Supermix (Bio-Rad, USA), and a BioRad CFX C1000 System. Details of qPCR procedures are provided in the Supporting Information.

### Assessment of Microbial Viability

In experiments with rNAu-1, the feedwater was deoxygenated prior to use, and changes in cell viability in response to deoxygenation were assessed. Two aliquots of freshly collected feedwater were tested in triplicate. One aliquot was stored at 4 °C, the other was deoxygenated, and both samples were treated with propidium monoazide (PMA) to assess the viability status of bacteria.^39^ DNA was subsequently extracted, and qPCR was performed for the 16S rRNA gene. Absolute concentrations of 16S rRNA in the deoxygenated samples ([9.28 ± 3.49] × 10^5^ copies/mL) were not significantly different (*p* = 0.2) than in the aerobic samples ([7.37 ± 0.87] × 10^5^ copies/mL), indicating that cell viability was not impacted by deoxygenation (see Figure S1).

### Data Processing and Statistical Analysis

All qPCR assay data were statistically analyzed with R Studio (version 3.5.2, http://www.r-project.org/). Gene removals were determined from the absolute gene concentrations in the feedwater before (*C*_feedwater_) and after each AOP treatment (*C*_AOP_) and are expressed in log_10_ units (eq 1)
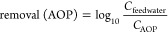
1qPCR data were statistically tested by pairwise comparison using the Games–Howell post hoc test with a significance cutoff of α = 0.05 (i.e., outcomes with *p* < 0.05 are statistically significant unless otherwise stated). Details of the statistical analysis are in the Supporting Information.

## Results and Discussion

### Benchmarking ARG Gene Removal by H_2_O_2_/NAu-1 against Conventional AOPs

We performed initial benchmarking experiments to assess whether activation of H_2_O_2_ by the natural iron-bearing clay mineral NAu-1 (H_2_O_2_/NAu-1) could enhance the destruction of target genes compared to UV irradiation and other AOPs (e.g., H_2_O_2_/UV). As the goal was to determine the potential of mineral-based AOPs for tertiary wastewater treatment, a subset of genes was trialed here to assess generic treatment efficiency for selected ARGs (*bla*_OXA-10_, *tet*M, and *tet*Q), bacteria (16S rRNA gene), and an example mobile genetic element cassette, MGE (*int*1). These genes (ARGs, MGE) are commonly associated with mechanisms of resistance to antibiotics^40−48^ that are frequently found in wastewater, and *tet*M was the most abundant ARG detected in the WWTP effluent used in our experiments as feedwater.

**Figure 1:**
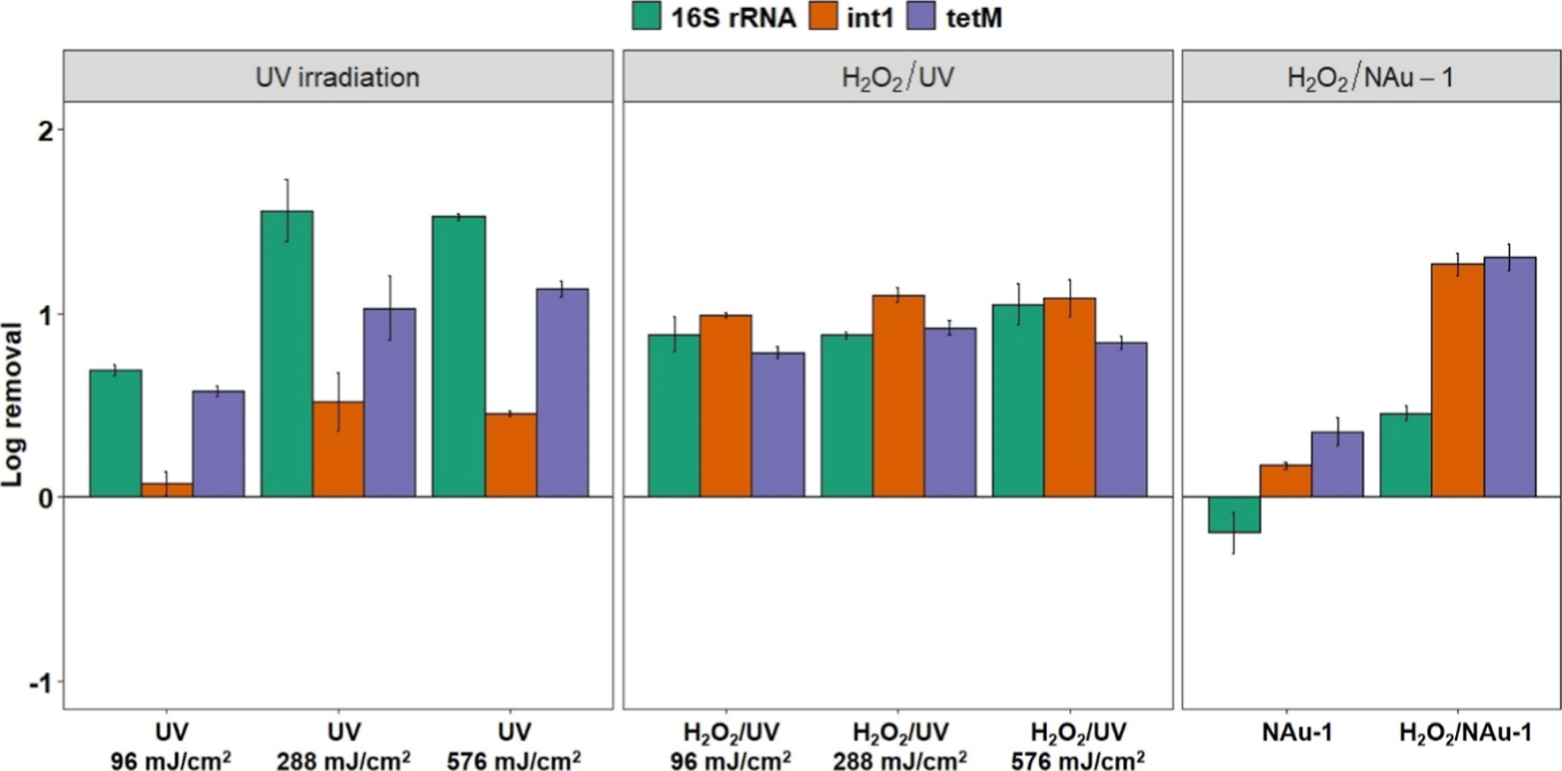
Log removal of genes 16S rRNA, *int*1, and *tet*M from secondary clarifier effluent (“feedwater”) after treatment with UV irradiation, H_2_O_2_/UV, or H_2_O_2_/NAu-1. Treatment conditions: 20 mM H_2_O_2_, 1 g/L NAu-1, 8 h contact time. Error bars indicate standard deviations from the mean of three replicate experiments.

Consistent with previous work,^9,49^ UV irradiation readily reduced the levels of target genes (i.e., 16S rRNA, *int*1, and *tet*M; Figure 1), and removal depended on UV dose. Increasing the UV dose from 96 to 576 mJ/cm^2^ increased the removals of 16S rRNA and *tet*M by 0.83 and 0.55 log units to a maximum of 1.52 and 1.13, respectively (Figure 1 and Table S3), which is similar to ranges observed previously (0.5–2.74 log units).^9,50^ Only the highest UV dose of 576 mJ/cm^2^ had statistically significantly greater removals relative to lower UV doses (Table S4), but the trends confirm that gene destruction is UV dose-dependent.^9,50^ Conversely, *int*1 removal was always less than 0.52 log units with UV irradiation (Figure 1 and Table S3), and no significant differences in removals were seen between UV doses (Table S4). Such differences in removals among target genes have been seen before^51^ and are believed to be related to the mode of action of UV irradiation. Interactions with nucleic acid molecules cause DNA damage, which affects different organisms differently,^52^ possibly due to variations in an organism’s ability of DNA repair.^53,54^

Adding 20 mM H_2_O_2_ with progressively increasing UV doses resulted in small but not statistically significant (typically *p* > 0.4) increases in gene removals (0.89 to 1.05, 0.99 to 1.10, and 0.79 to 0.92 for 16S rRNA, *int*1, and *tet*M genes, respectively; Figure 1 and Table S3). Gene removal with H_2_O_2_ addition did not appear to be enhanced by UV irradiation, even at higher UV doses. This is possibly because of light attenuation and the scavenging effects of elevated organic matter content in the wastewater matrix^31,55^ and/or the H_2_O_2_ dose^56^ being rate-limiting under our combined H_2_O_2_/UV treatment conditions.

However, H_2_O_2_ addition itself did significantly increase *tet*M gene removal at a conventional UV dose^11^ of 96 mJ/cm^2^ and increased *int*1 removal for all UV doses compared with UV irradiation alone (Figure 1 and Table S4), indicating that combining H_2_O_2_ and UV was generally more effective than UV alone. In theory, adding H_2_O_2_ in tandem with UV irradiation should enhance gene destruction, with UV penetrating the cytoplasm and damaging DNA, and reactive oxygen species (ROS) from H_2_O_2_ decomposition enhancing gene damage.^51,54^ Similar removals for all three genes (Figure 1 and Table S3) suggest that more reactive but less selective ROS were the main cause of gene removal in the combined H_2_O_2_/UV treatment.

With this background, we assessed whether iron-bearing clay mineral NAu-1 could catalyze ROS production from H_2_O_2_, which would be consistent with H_2_O_2_ activation by other iron-bearing silicate minerals.^14,16^ We hypothesized that increased nonselective gene removal will occur with NAu-1 addition. As expected, NAu-1 (1 g/L) alone had little effect on *tet*M and *int*1 gene removals. In fact, NAu-1 slightly increased 16S rRNA gene levels (Figure 1), probably due to new bacterial growth in the presence of the clay mineral.^26,57−59^

In contrast, addition of H_2_O_2_ combined with NAu-1 significantly increased 16S rRNA, *int*1, and *tet*M gene removals (log values 0.46, 1.27, and 1.31, respectively; *p* < 0.01; Figure 1 and Tables S3 and S4). Although H_2_O_2_/NAu-1 displayed a lower removal of 16S rRNA genes compared to H_2_O_2_/UV and UV alone, combining H_2_O_2_ with NAu-1 significantly increased *int*1 and *tet*M gene removals (*p* ≤ 0.02, Table S4), with the highest observed removals across all treatments (Figure 1). Differences in removals between *int*1 and *tet*M versus 16S rRNA genes imply that combined H_2_O_2_/NAu-1 treatment could be more selective than H_2_O_2_/UV treatment without NAu-1, although both treatments clearly form ROS as their active components.^13,14^ To further examine NAu-1 addition for practical applications, we assessed the effect of other treatment parameters on ARG removal, specifically the H_2_O_2_ dose and contact time.

### Activation of H_2_O_2_ Using NAu-1: Effect of Treatment Parameters

**Figure 2:**
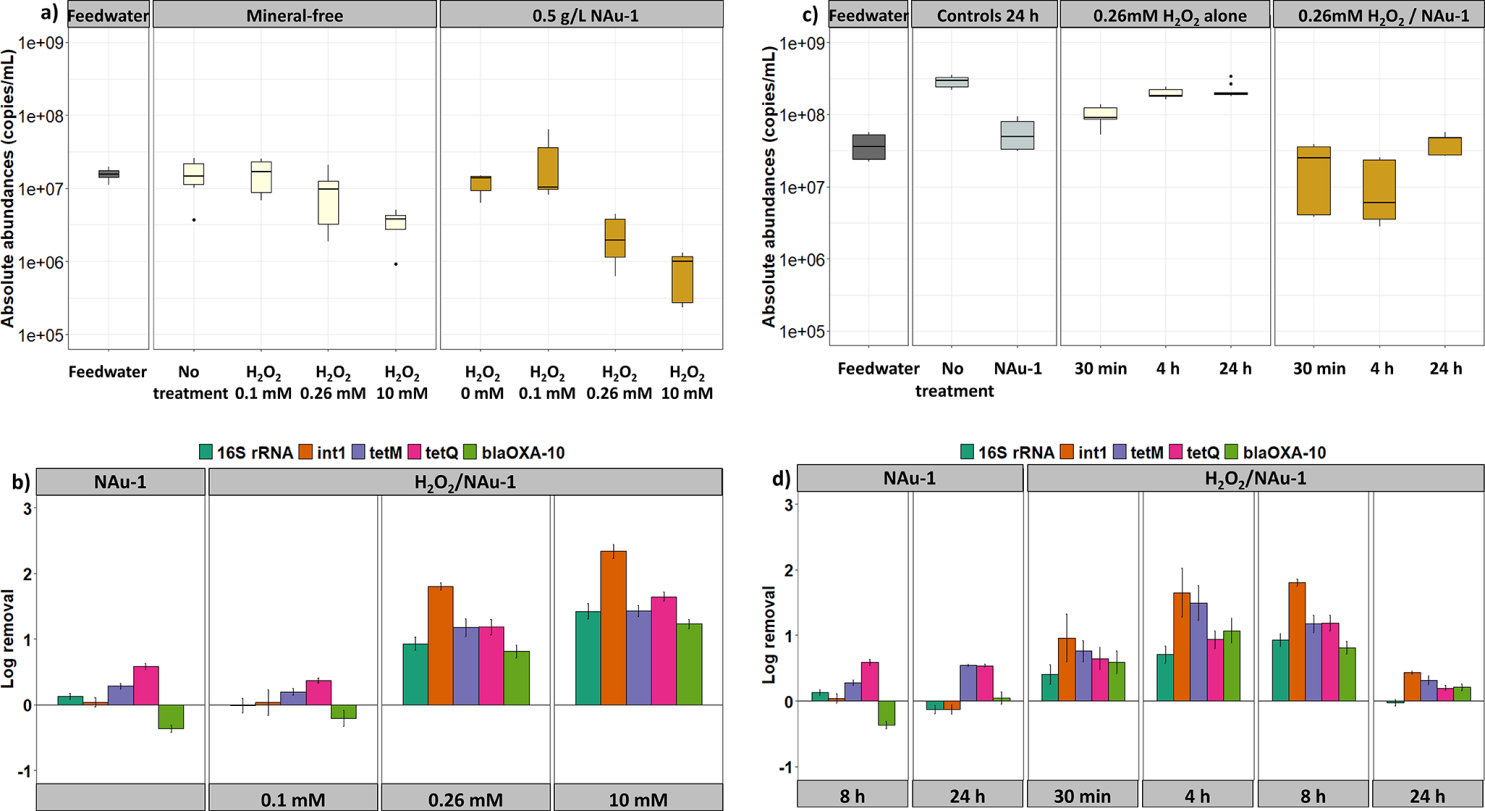
Effect of treatment parameters (a,b) H_2_O_2_ dose and (c,d) contact time on (a,c) absolute abundances of 16S rRNA (gene copies/mL) and (b,d) on the removals of target genes during treatment with H_2_O_2_ combined with iron-containing clay mineral NAu-1. Controls include feedwater alone stirred at 22 ± 2 °C (“No treatment”), addition of different concentrations of H_2_O_2_ alone, and addition of NAu-1 alone. Error bars in (b,d) indicate standard deviations from the mean of three replicate experiments. Reaction conditions: (a,b) 8 h contact time, 0.5 g/L NAu-1, (c,d) 0.26 mM H_2_O_2_, 0.5 g/L NAu-1.

In experiments to assess different H_2_O_2_ doses (0.1, 0.26, and 10 mM) and contact times (30 min, 4 h, 8 h, and 24 h) in the H_2_O_2_/NAu-1 treatment, the concentration of NAu-1 was reduced to 0.5 g/L to reduce clay mineral input to the treatment process. In these experiments, the pool of target genes was expanded to include *bla*_OXA-10_ and *tet*Q^60^ as well as the 16S rRNA gene, *int*1, and *tet*M. Because similar reductions in absolute abundances were seen among all genes tested (Tables S5 and S6), only 16S rRNA gene data will be used here to show the trends relative to H_2_O_2_ dose and contact time (Figure 2a,c).

Initial control experiments without addition of H_2_O_2_ or NAu-1 (“no treatment”) or with addition of only NAu-1 showed no statistically significant changes in absolute abundances over 8 h of contact time (Table S7) relative to the initial feedwater (Figure 2a). Addition of 0.1 mM H_2_O_2_ alone also had no significant effect on the absolute 16S rRNA gene abundances over time. However, increasing the H_2_O_2_ dose did reduce gene abundances (Figure 2a), although reductions were only statistically significant at the highest H_2_O_2_ dose (10 mM, Table S7). These results confirm that very high levels of H_2_O_2_ are needed to achieve even moderate (<1 log unit) gene removals when H_2_O_2_ is the sole oxidant.^61^

However, over a longer contact time of 24 h, significant increases in 16S rRNA gene abundances were observed in the reactors (*p* < 0.01, Figure 2c and Table S8), suggesting some new bacterial growth occurred later in the experiments. Addition of NAu-1 alone did inhibit new growth, as indicated by nonsignificant differences in absolute 16S rRNA gene abundances between the feedwater and NAu-1 alone (*p* = 0.77, Table S8). These results suggest that the addition of clay mineral can have a bacteriostatic effect. We suggest that the lower clay mineral load (0.5 g/L) compared to the benchmarking experiments could be responsible for this apparent change in the clay mineral effect from growth-promoting to bacteriostatic. In contrast, addition of H_2_O_2_ alone had no such effect—subsequent 16S rRNA gene abundances increased with 24 h of contact time (Figure 2c).

When H_2_O_2_ and NAu-1 were combined, absolute gene abundances were reduced for all contact times (Figure 2c and Table S6) and for the 0.26 and 10 m H_2_O_2_ doses (Figure 2a and Table S5). However, 0.1 mM H_2_O_2_ with 0.5 g/L NAu-1 failed to reduce 16S rRNA gene abundances compared to controls (feedwater, 0.1 mM H_2_O_2_ alone, and NAu-1 alone). These results suggest a minimum effective H_2_O_2_ dose is needed to obtain significant reductions in gene abundances, and in our reactors, this dose is somewhere between 0.1 mM and 0.26 mM H_2_O_2_. Moreover, if one compares 16S rRNA gene removals between our moderate vs highest H_2_O_2_ dose (Figure 2b and Table S9), significant differences are not seen (Table S10), suggesting that a 0.26 mM H_2_O_2_ dose in the presence of clay mineral NAu-1 may be pseudo-optimal for gene removal.

**Figure 3:**
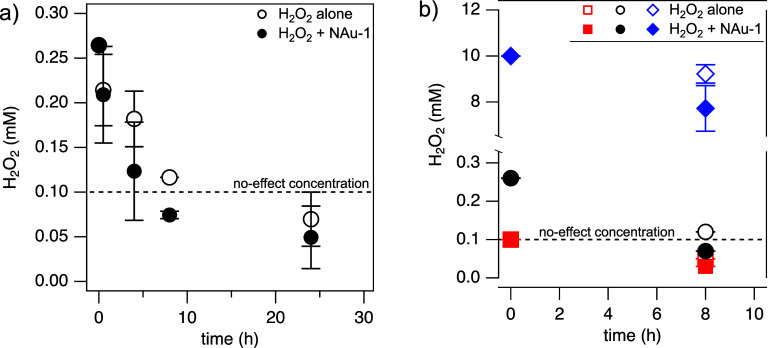
H_2_O_2_ concentrations (mM) added to secondary clarifier effluent in the absence (open markers; H_2_O_2_ alone) and presence of 0.5 g/L NAu-1 (filled markers; H_2_O_2_ + NAu-1), monitored for (A) different contact times with a constant initial H_2_O_2_ dose of 0.26 mM and (B) as a function of initial H_2_O_2_ dose (square: 0.1 mM, circle: 0.25 mM, diamond: 10 mM) after 8 h of contact time. The black dashed line indicates the H_2_O_2_ concentration, which needs to be exceeded to observe significant gene removal (see Figure 2b). Error bars represent standard deviations from the mean of three replicate experiments.

Relative to contact time, changes in 16S rRNA gene abundances were not significantly different between contact times of 30 min and 4 h (Table S8), whereas an 8 h contact time resulted in significant reductions in gene levels (Figure 2d). However, with a 24 h contact time, 16S rRNA gene abundances increased significantly (*p* < 0.01, Table S8), suggesting bacteria regrowth can occur if contact time is too long, presumably due to the depletion of oxidant. In fact, residual H_2_O_2_ concentrations decreased with contact time to levels below the minimum effective H_2_O_2_ concentration (Figure 3), which explains a pseudo-optimal contact time between 4 and 8 h in our system. We suspect that this time will differ if different H_2_O_2_ and NAu-1 doses are used. At our pseudo-optimal H_2_O_2_/NAu-1 treatment conditions (4–8 h, 0.26 mM H_2_O_2_), 16S rRNA gene removal rates of 0.71–0.93 were observed and were significantly higher than in the controls, H_2_O_2_ only additions, and contact times (Figure 2d and Tables S9–S11). At the same time, H_2_O_2_ activation exceeded that in the absence of NAu-1, and residual H_2_O_2_ concentrations remained above or close to the dose that was found to be ineffective (0.1 mM, Figure 3). The combination of results suggests that ROS, such as •OH, formed during the reaction of H_2_O_2_ with clay Fe(III)^16,62^ and points to a bactericidal effect of the combined H_2_O_2_/NAu-1 treatment.^16,62^

In contrast, at our longest contact time (24 h), residual H_2_O_2_ concentrations fell to even lower values (Figure 3), and we observed a bacteriostatic effect of NAu-1 (Figure 2d). Previous work linked the bacteriostatic effects of clay minerals to their modulation of pH to acidic (pH < 5) or alkaline (pH > 9) conditions and/or the release of toxic metals.^63^ In our experiments, the pH value increased from 6.8 to a maximum of 8.3 (Figure S2) when NAu-1 was added. Such a final pH value should not impact bacterial growth^64^ nor would it induce metal release from the clay mineral.^63^ Indeed, metal analysis indicated slightly lower dissolved Fe(II) and total Fe concentrations in the reactors after treatment (Table S12), confirming that metal release probably did not cause a bacteriostatic effect. We speculate that the bacteriostatic effect of NAu-1 may rather be attributed to physical interactions between the clay mineral and bacterial membranes, impairing bacterial physiological function.^63,65^

### Differences between Removals of the 16S rRNA Gene and the ARGs and MGE

Although the other target genes displayed trends similar to those of the 16S rRNA data, two small but relevant deviations from these general trends were seen for different genes (Figure 2b,d and Table S9). First, increasing the H_2_O_2_ dose in the H_2_O_2_/NAu-1 treatment from 0.1 mM to 0.26 and 10 mM resulted in significantly higher removals for all genes (*p* ≤ 0.03, Table S10), ranging from 0.81 to 2.24 (Figure 2b). The highest H_2_O_2_ dose achieved significantly higher removals for *int*1 and *bla*_OXA-10_, whereas removals were greater, yet not statistically different, for the 16S rRNA, *tet*M, and *tet*Q genes when compared to a dose of 0.26 mM (*p* > 0.07, Table S10). These data suggest that moderate H_2_O_2_ doses in the H_2_O_2_/NAu-1 treatment can reduce diverse genes, and higher removals may not be consistently achieved with higher H_2_O_2_ doses.

Second, among the target genes, *int*1 consistently displayed the highest removals at the effective H_2_O_2_ doses (0.26, 10 mM; Figure 2b) at all contact times (Figure 2d and Table S9) among the three genes of similar amplicon size (*int*1:196 bp, *tet*Q: 167 bp, and *bla*_OXA-10_: 191 bp), albeit differences were not always statistically significant (Table S13). Direct comparisons with 16S rRNA and *tet*M removals were not possible due to larger and smaller, respectively, amplicon target sequences. However, differences among the similar amplicon sequence genes suggest some selectivity in H_2_O_2_/NAu-1 treatments, as hypothesized from our benchmarking experiments. We suspect the potentially greater presence of *int*1 outside bacterial cells (e.g., as part of plasmids)^31^ could make this gene more susceptible to extracellular oxidants formed.^66,67^ Consistent with this known trait of MGEs, higher relative gene abundances of *int*1 (>10 times) were observed compared to the ARGs in all reactors (Figures S3 and S4). Consequently, significantly different removals and patterns for *int*1 on the one side and *bla*_OXA-10_ and *tet*Q on the other side suggest some ARGs may primarily be removed by killing their bacterial hosts in the H_2_O_2_/NAu-1 treatments.

### Effect of H_2_O_2_/NAu-1 Treatment on Other Wastewater Constituents

We also evaluated how H_2_O_2_/NAu-1 treatment affected the wastewater physicochemical parameters pH, COD, TP, TSS, and TOC (Table S14). Treatments under pseudo-optimal conditions (0.26 H_2_O_2_, 0.5 g/L NAu-1, 4, and 8 h contact times; Figure 2) slightly reduced COD and TP by 6–7 (±2–3)% and 25–27 (±2)%, respectively. Both TSS and TOC slightly increased, which we suspect is due to the release of bacterial cell debris during the oxidative treatment.^68,69^ The pH value became more alkaline as a result of H_2_O_2_ decomposition^16^ but still remained below 8, which is within the range normally observed in natural and engineered ecosystems.^70^

Because the mineral structure of NAu-1 contains Fe, which is the active catalytic site for H_2_O_2_ activation,^16^ we monitored the potential release of Fe into the reactor aqueous phase. During H_2_O_2_/NAu-1 treatment, total aqueous Fe concentrations decreased (Table S12), showing that NAu-1 did not release Fe from its structure as part of the treatment and rather appears to be a sink for metal ions. Hence, the same clay mineral could potentially be reused in subsequent treatment cycles for H_2_O_2_ activation, although this must be assessed in future studies.

### Treatment Using Reduced NAu-1

**Figure 4:**
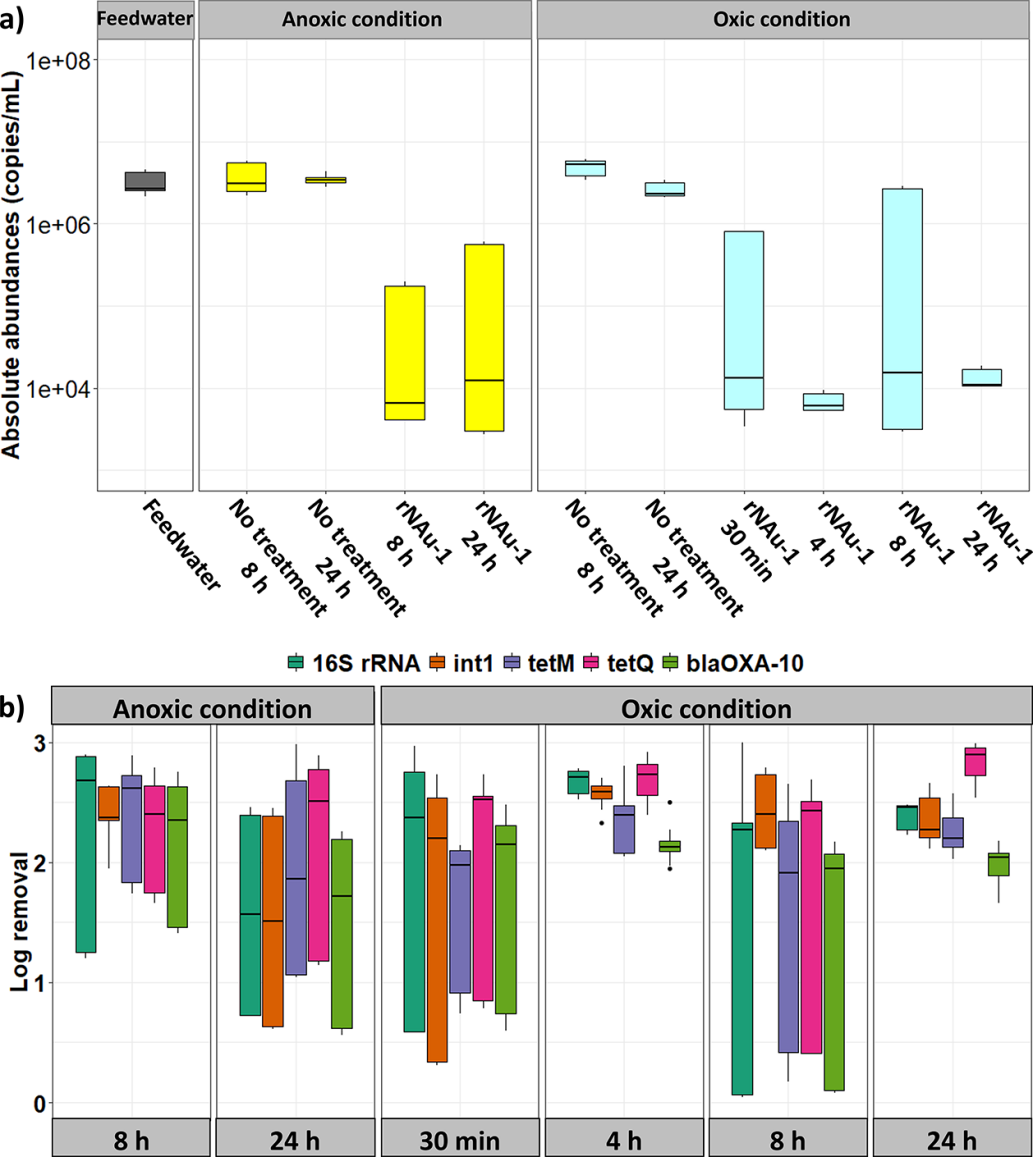
(a) Absolute abundances of the 16S rRNA gene (gene copies per mL) in the presence and absence of 0.5 g/L reduced clay mineral NAu-1 (rNAu-1) under anoxic conditions (anaerobic glovebox with <2 ppm of O_2_) and oxic conditions (dark room with ambient O_2_ levels). “No treatment” refers to deoxygenated feedwater stirred at 22 ± 2 °C in the absence of rNAu-1. (b) Removals (log values) of all target genes when in contact with rNAu-1 for different durations under anoxic and oxic conditions.

Finally, we tested our hypothesis that H_2_O_2_ and other ROS produced in situ from reduced (i.e., Fe(II)-containing) NAu-1 upon contact with O_2_^19,20^ may also effectively remove genes from the WWTP secondary effluent. Gene removals were compared in the presence of reduced NAu-1 (rNAu-1) under anoxic (glovebox with <2 ppm of O_2_) versus oxic conditions (dark room with ambient O_2_ levels). Experiments were undertaken using the same NAu-1 concentrations as before (0.5 g/L) for 30 min and 4, 8, and 24 h contact times. Data for the 16S rRNA gene are used again to exemplify overall trends (Figure 4a and Table S15).

**Figure 5:**
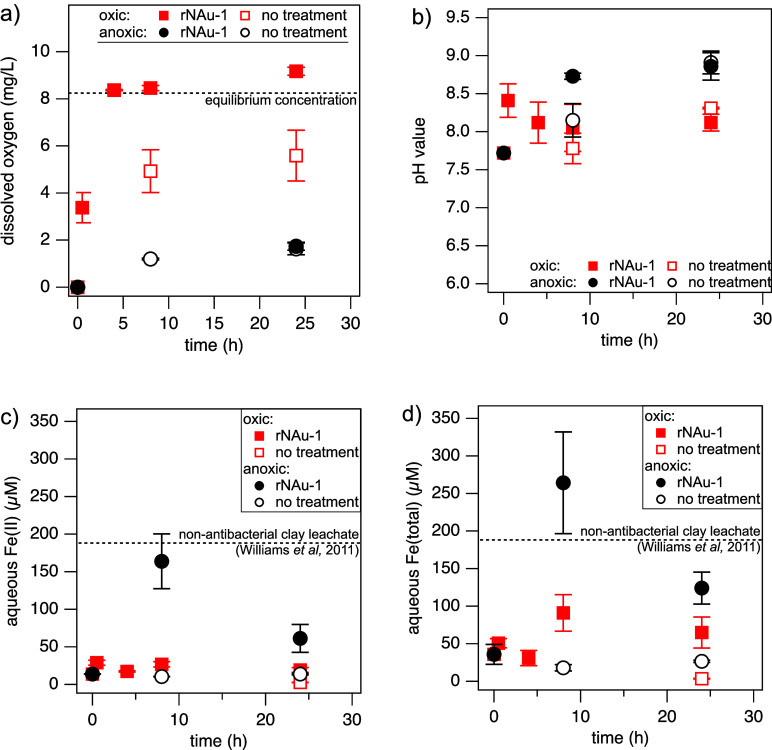
Aqueous phase parameters: (a) dissolved oxygen (DO) concentrations, (b) pH values, (c) dissolved Fe(II) concentration, and (d) dissolved total Fe concentration over time in deoxygenated secondary clarifier effluent alone (“no treatment”: open markers) and with the addition of rNAu-1 (filled markers) under oxic (red squares) and anoxic conditions (black circles). Error bars represent standard deviations from the mean of three replicate experiments. The dashed line in (a) indicates the DO concentration at equilibrium with oxygen in air, 8.24 mg/L (*T* = 25 °C), and the dashed lines in (c,d) indicate the aqueous Fe concentration in leachates from non-antibacterial clay at similar experimental conditions (pH 8).^26^

Without rNAu-1 added (i.e., “no treatment”), 16S rRNA gene abundances over 8 and 24 h contact time displayed no change under both oxic and anoxic conditions (Figure 4a and Table S16), suggesting possible bacteriostatic effects due to deoxygenation. Even though deoxygenation did not appear to affect bacterial viability (Figure S1), such conditions can potentially deprive many bacteria of their preferred electron acceptor, dissolved oxygen^71^ (Figure 5a), and slow down bacterial growth. In contrast, addition of rNAu-1 under oxic conditions strongly (>2 orders of magnitude) and significantly decreased 16S rRNA gene abundances (Figure 4a, *p* ≤ 0.03). Removals exceeded those of the H_2_O_2_/NAu-1 treatment (Figure 2) and are consistent with previous reports of antibacterial effects on *E. coli* upon exposure to natural clays^25^ or to reduced clay mineral NAu-2 (structurally highly similar to NAu-1) under oxic conditions.^27^

Abundances of all other genes (*int*1, *bla*_OXA-10_, *tet*M, and *tet*Q) were also significantly reduced when in contact with rNAu-1 under oxic conditions (Tables S15 and S16), with *bla*_OXA-10_ abundances falling below the limit of quantification after 24 h (LoQ: 17 copies/mL; Table S2). Removals were greatest for contact times of 4 and 24 h (log values of 1.98–2.94, Figure 4b and Table S17), yet differences across contact times were not statistically significant (*p* > 0.06, Table S18). This suggests that rapid (i.e., within 30 min), substantial (>1.5 log unit), and nonselective gene removal is possible during oxidative treatment with rNAu-1.

Several possible mechanistic explanations for the antimicrobial effects of Fe(II)-containing clay minerals have been discussed in previous studies. One proposed mechanism is that Fe(II) is released from the clay mineral due to suspension pH alterations and is taken up by bacteria, where subsequent intracellular Fenton processes induce oxidative damage.^25,63^ An alternative explanation is that the oxidation of clay mineral Fe(II) forms extracellular ROS, particularly •OH, which might lead to microbial cardiolipin damage and cell death.^27^ In our experiments with rNAu-1, both pH value (8.0–8.4, Figure 5b) and aqueous Fe(II) concentrations (44–72 μM, Figure 5c) increased, pointing toward the first mechanism. However, a similar change in pH values during the NAu-1/H_2_O_2_ treatment (Figure S2) did not alter the aqueous Fe concentrations (Table S12). Our data imply that rather than a pH alteration, the presence of organic material (TOC = 10 mg/L, Table S14) caused Fe(II) and Fe(III) release from the reduced clay mineral via complex formation.^72^ Moreover, resulting aqueous Fe concentrations remained well below those of non-antibacterial clay leachates (>150 μM),^25^ and aqueous Al concentrations were below the quantification limit (0.89 μM), suggesting that bacterial uptake of released metal ions^73^ was unlikely responsible for gene removal here.

A more plausible mechanism is based on our observation that the extent of clay mineral Fe reduction (i.e., Fe(II)/Fe(total)) decreased with reaction time from initially 80% to 34% after 24 h (Table 1). These results indicate that clay mineral Fe(II) became oxidized, which may have led to the formation of ROS, such as •OH, and the destruction of cell material.^27^ Consistent with substantial gene removals after short contact time (Figure 4b), more than half of the total clay mineral Fe(II) oxidation (and hence ROS formation) occurred already within the first 30 min (Table 1), and both total clay mineral Fe(II) oxidation and gene removals increased with longer contact times. Our finding of 16S rRNA gene removals of 1.8 to 2.7 log units (Figure 4b and Table S17), however, contrasts with previous reports of the survival of *E. coli* under similar conditions.^27^ Concentrations of •OH measured during the oxygenation of rNAu-2 at relevant pH values (pH 7–8)^27^ were 40–60 μM, which is only slightly lower than the calculated stoichiometric •OH yields in our experiments (150–270 μM, Table 1). We suspect that the presence of organic matter in our experiments may have enhanced degradation reactions with bacteria and genes, similar to •OH yield enhancements found for the oxygenation of Fe(II)-containing (clay) minerals in the presence of small organic and humic acids.^72,74^

**Table 1:** Calculations of Fe(II) Mass Balance and Theoretical Maximum OH Radical (•OH) Yield as a Function of Contact Time in rNAu-1 Treatement Under Both Oxic and Anoxic Conditions^a^

	oxic conditions						anoxic conditions					
	clay mineral Fe		aqueous Fe	total Fe^b^	oxidation		clay mineral Fe		aqueous Fe	total Fe^b^	oxidation	
time	Fe(II)/Fe(total)^c^	Fe(II)^d^	Fe(II)	Fe(II)	Fe(II)^e^	•OH yield^f^	Fe(II)/Fe(total)^c^	Fe(II)^g^	Fe(II)	Fe(II)	Fe(II)^e^	•OH yield^f^
h	%	μM	μM	μM	μM	μM	%	μM	μM	μM	μM	μM
0	80.3 ± 0.7^h^	1423 ± 12	13.8 ± 0.0	1437 ± 12			80.3 ± 0.7^h^	1423 ± 12	13.8 ± 0.0	1437 ± 12		
0.5	54.8 ± 0.2	972 ± 4	28.9 ± 3.3	1001 ± 5	437 ± 14	146 ± 5						
4	40.7 ± 0.2	721 ± 4	17.4 ± 0.9	738 ± 4	699 ± 13	233 ± 4						
8	39.7 ± 0.4	709 ± 7	26.8 ± 3.6	735 ± 8	702 ± 15	234 ± 5	65.8 ± 0.3	1068 ± 259	164 ± 36	1232 ± 262	205 ± 262	68 ± 87
24	34.3 ± 0.4	607 ± 7	19.2 ± 3.2	626 ± 8	811 ± 15	270 ± 5	64.4 ± 0.6	1111 ± 437	61 ± 19	1172 ± 437	265 ± 437	88 ± 146

a Standard deviations from the mean of three replicate experiments are provided.

b Total Fe in the reactors was calculated for Fe(II) only, as the sum of the concentrations of Fe(II) in clay mineral NAu-1 and the aqueous phase.

c Clay mineral Fe reduction extent was determined from the relative spectral area of the Fe(II) doublet in the samples’ Mössbauer spectra collected at 77 K.

d For experiments in oxic conditions, clay mineral Fe(II) concentrations were calculated using the suspension’s mass loading (0.5 g/L), the measured total Fe content of clay mineral NAu-1 (19.8 wt %), and the clay mineral Fe reduction extent. Release of mineral Fe(II) to the aqueous phase was negligible and hence not corrected for.

e Oxidised Fe(II) is the difference between the initial total Fe(II) concentration (*t* = 0 h) and at a given time point during the experiment (*t* > 0 h).

f The theoretical maximum yield of •OH produced from the reaction of (clay mineral) Fe(II) with dissolved oxygen was calculated using the concentration of Fe(II) oxidized and the reaction’s stoichiometry of Fe(II):O_2_ of 3:1.^21^

g For experiments in anoxic conditions, clay mineral Fe(II) concentrations were calculated using the suspension’s mass loading (0.5 g/L), the measured total Fe content of clay mineral NAu-1 (19.8 wt %), and the clay mineral Fe reduction extent and are corrected for the release of mineral Fe(II) to the aqueous phase.

h The reduction extent before the beginning of the experiments (*t* = 0) was determined photometrically using the 1,10-phenanthroline method after HF digestion of rNAu-1.

Surprisingly, addition of rNAu-1 to deoxygenated feedwater under anoxic conditions (O_2_ < 2 ppm) also reduced the absolute abundances of all target genes after 8 and 24 h of contact time (Figure 4a), and both gene abundances (*p* > 0.68) and removals were not statistically different than under oxic conditions (Figure 4b, *p* > 0.35). Our results contrast with a previous report that *E. coli* abundances were minimally affected by the presence of reduced clay mineral under anoxic conditions,^27^ implying that the mechanisms of the bactericidal effect of rNAu-1 under anoxic vs oxic conditions differ between studies.

Indeed, DO concentrations remained low under anoxic conditions (≤1.74 mg/L, Figure 5a) and were limited by the glovebox atmosphere O_2_ concentrations (<2 ppm). These data confirm that gene removal with rNAu-1 under anoxic conditions was unlikely caused by accidental oxygenation, which may have led to ROS formation. The much higher aqueous Fe(II) concentrations in anoxic reactors (60–165 μM, Figure 5c) compared to oxic conditions rather point to the potential for increased uptake of aqueous Fe(II) by bacteria and subsequent intracellular ROS production^25^ as a more plausible mechanism for gene removal.

The release of Fe(II) from the clay mineral is also reflected in the, at first sight, unexpected decrease in clay mineral Fe reduction extent under anoxic conditions. The final Fe(II)/Fe(total) ratios (64–66%) remained substantially higher compared to oxic conditions (34%) and equate to only 205–265 μM of Fe(II) oxidized when considering the reactor Fe(II) mass balances (Table 1). We cannot rule out the possibility that oxidation of even these limited amounts of Fe(II) was sufficient to exert a bactericidal effect. Within error, released Fe(II) accounts for the apparent clay mineral Fe(II) oxidation, and hence, no net oxidation and ROS formation occurred in the anoxic reactors. We suspect that the organic matter (TOC: 10 mg/L) in the wastewater matrix enabled the release of Fe(II) from the clay mineral structure at these high pH values (∼8.8, Figure 5a) via complex formation. Yet how these Fe(II)-OM complexes might lead to bactericidal effects and gene removal must be explored in future research.

An alternative mechanism that could explain similar gene removal with rNAu-1 under oxic and anoxic conditions is the sorption of ARB and ARGs to the surface of clay mineral particles, which would render genes inaccessible to quantification in filtrates. However, sorption was not observed in our experiments with nonreduced NAu-1 (Figures 1 and 2) and was previously found to be irrelevant for natural and redox-activated clay minerals.^27,75,76^ Indeed, repulsive forces between clay mineral surfaces and bacteria have been reported,^77^ and reduction of Fe within NAu-1 increased the mineral’s negative excess charge,^78^ resulting in even greater repulsive forces. We therefore conclude that sorption is unlikely to explain our results from treatment with rNAu-1 and suggest that the two different modes of action of rNAu-1 under oxic vs anoxic conditions are more plausible mechanisms.

## Conclusions

This work provides proof-of-concept evidence that mineral-based AOPs have potential as low chemical and energy tertiary wastewater treatment options. Bacteria (as 16S rRNA gene), ARG, and MGE removals of up to 2.3 log units are possible with H_2_O_2_/NAu-1 treatment, which is similar to or better than UV irradiation and H_2_O_2_/UV treatment. Further, rNAu-1 addition achieved even higher gene removals (up to 3 log units), which is similar to common non-AOP treatment technologies, such as chlorination, reverse osmosis, or membrane filtration.^5^ However, all these traditional technologies demand substantial amounts of energy and chemicals and can be at least triple the cost of secondary treatment operations.^5^ In contrast, clay mineral Fe-reduction can be performed by native bacteria under oxygen-free conditions,^57^ such as in anaerobic digesters, without the need for additional chemicals or energy input. Further research is needed to operationalize “rNAu-1 tertiary treatment” from the lab to pilot to full scale, but huge potential savings are possible.

For the more conventional approach of activating H_2_O_2_, pseudo-optimal treatment conditions of 0.26 mM H_2_O_2_, 0.5 g/L NAu-1, and 4–8 h contact time for gene removal are provided here. Both H_2_O_2_ concentrations and contact times are within feasible ranges for use in full-scale WWTPs (0.05–8.37 mM H_2_O_2_),^35,61^ including activated sludge processes (hydraulic retention time: 0.57–5.4 d).^79^ Hence, the addition of this mineral-based AOP as a tertiary treatment step to conventional WWTPs should be feasible. Further, bacterial regrowth, a general problem in many tertiary/quaternary treatment approaches,^80,81^ might be reduced by the clay mineral’s bacteriostatic capability and/or dosing of H_2_O_2_ above the reactive levels identified here. Optimal H_2_O_2_ concentrations for effective treatment, which minimize chemical inputs and prevent disinfection byproduct formation,^82^ should be confirmed.

Finally, mineral-based oxidation reactions may also play a significant role in natural sedimentary environments, where clay minerals are ubiquitous.^83^ Most clay minerals contain some Fe in their structure (0–33 wt %)^25^ that is susceptible to microbial reduction under anoxic conditions.^84^ Our results of rapid (e.g., 30 min) and nonselective gene and bacteria removal by rNAu-1 suggest that reduced clay minerals may be promoted for in situ remediation and nature-inspired treatment systems, such as riverbank filtration or wetlands. Clearly, the results reported here are only a proof of concept, and we strongly encourage further work on mineral-based AOPs because of their diverse utility in many remediation applications.

## Supporting Information Available

Additional information on methods (wastewater sample characterization, Mössbauer spectroscopy, gene quantification, microbial viability assessment, and statistical analysis) and results (absolute abundances, log removals, outcomes of statistical analyses, and pH values, aqueous Fe content, and gene relative abundances in reactors with H_2_O_2_/NAu-1 treatment) (PDF)
