# Supplementary material for: Mineral-Based Advanced Oxidation Processes for Enhancing the Removal of Antibiotic Resistance Genes from Domestic Wastewater
Source: ACS ES T Water. 2025 Apr 29;5(5):2310–21. doi: 10.1021/acsestwater.4c01213 (PMC12070418; doi:10.1021/acsestwater.4c01213)
Supplement: Supplementary file 1 — ew4c01213_si_001.pdf [file ew4c01213_si_001.pdf]

Supporting Information

Mineral-based advanced oxidation processes for enhancing the removal of antibiotic resistance genes from domestic wastewater

Panagiota Adamou[1], James Entwistle[1], David W. Graham[1], Anke Neumann*[1,2]

[1]School of Engineering, Newcastle University, Newcastle upon Tyne, NE1 7RU, United Kingdom; [2] PSI Center for Nuclear Engineering and Sciences, 5232 Villigen PSI, Switzerland

*corresponding author email address: anke.neumann-jenal@psi.ch

1. METHODS 2

1.1. WASTEWATER SAMPLE CHARACTERISATION 2
1.2. MÖSSBAUER SPECTROSCOPY 2
1.3. GENE QUANTIFICATION 2
1.4. ASSESSMENT OF MICROBIAL VIABILITY 3
1.5. STATISTICAL ANALYSIS 4

2. RESULTS 4

2.1. BENCHMARKING ARG GENE REMOVAL BY H_2O_2/NAU-1 AGAINST CONVENTIONAL AOPS 4
2.2. ACTIVATION OF H_2O_2 USING NAU-1: EFFECT OF TREATMENT PARAMETERS 6
2.3. TREATMENT USING REDUCED NAU-1 18

1. METHODS

1.1. WASTEWATER SAMPLE CHARACTERISATION

Table S1. Water quality characterisation of the secondary clarifier effluent from a domestic WWTP. Values are averages (± standard deviation) of measurements taken on a total of 4 samples collected at separate sampling days and for individual experimental sets. (DO: dissolved oxygen; COD: chemical oxygen demand; TSS: total suspended solids)

**DO** mg/L	**Conductivity** µS/cm	**COD** mg/L	**TSS** mg/L	**pH** -
2.5 ± 0.3	733 ± 65	27.4 ± 8.5	24.2 ± 7.0	6.4 ± 0.3

1.2. MÖSSBAUER SPECTROSCOPY

Solid samples were analysed on a MS4 Mössbauer spectrometer (SEE Co., Edina, MN) equipped with a closed-cycle cryostat (SHI-850, Janis Research Co., Wilmington, MA). Spectra were collected in transmission geometry using a Co-57 source in a Rh matrix in constant acceleration mode and calibrated against α-Fe(0) foil. To enable Fe(II)/Fe(total) content determination in the Fe-rich clay mineral[1], spectra were collected at 77 K. The spectra were analysed using the Voigt-based fitting routine[2] implemented in the software package Recoil (Ottawa, Canada)[3].

1.3. GENE QUANTIFICATION

Quantification of genes was performed by quantitative polymerase chain reaction (qPCR) on a BioRad CFX C1000 System (BioRad, Hercules, CA, USA). Details of the specific primers used to amplify the target genes (16S rRNA, *int*1, bla_{OXA-10}, *tet*M and *tet*Q) are given in Table **S2**. For quantification of all genes, 2 µL template DNA was used in a reaction mixture containing 7.5 µL SsoFastTM EvaGreen® Supermix (Bio-Rad, USA), 300 nmol/L of each forward and reverse primer (Thermo Fisher Scientific, UK), and H_2O to a final volume of 15 µL. Reaction conditions for gene quantification included an initial denaturing step at 98 °C for 2 min, followed by 40 cycles with each cycle consisting of denaturation at 98 °C for 5 s and annealing temperature at 60 °C for 30 s. Each sample was amplified in triplicate and H_2O replaced template in control reactions to check any contamination. To avoid amplification inhibition due to the potential presence of humic acids or other impurities in the samples, DNA templates were diluted to a working solution of 5 ng/µL. Standards for each specific gene were produced from sequenced genes from environmental samples.

Standards exponentially increase between 10 and 35 quantification cycles (Cq), thus, any standard with Cq > 35 were considered unreliable[4]. Based on the lowest accurate standard on the curves, which was gene-specific, the limits of quantification (LoQ) were 2.53 copies/mL (Cq = 34.4), 22.5 copies/mL (Cq = 34.5), 16.9 copies/mL (Cq = 35), 218 copies/mL (Cq = 32.4) and 2.48 copies/mL (Cq = 34.1) for the 16S rRNA, *int*1, bla_{OXA-10}, *tet*M and *tet*Q, respectively.

Table S2. Gene targets, primers and their details (sequence, base-pairs/amplicon size), and references describing their amplification as used in this study. Limits of quantification (LoQ) specific to this study are also presented.

Target	Primer	Primer sequence (5'-3')	Base-pairs	Reference	LoQ (copies/mL)
16S rRNA	1055 F	ATGGCTGTCGTCAGCT	337	Harms et al.[5]	2.53
	1392 R	ACGGGCGGTGTGTAC			
*tet*Q	*tet*Q F	AGAATCTGCTGTTTGCCAGTG	167	Wang et al.[6]	2.48
	*tet*Q R	CGGAGTGTCAATGATATTGCA			
bla$_{OXA-10}$	*bla*$_{OXA-10}$ F	AGAGGCTTTGGTAACGGAGG	191	Wang et al.[6]	16.9
	bla$_{OXA-10}$ R	TGGATTTTCTTAGCGGCAAC			
*tet*M	*tet*M F	GGTTTCTCTTGGATACTTAAATCAATCR	88	Peak et al.[7]	218
	*tet*M R	CCAACCATAYAATCCTTGTTCRC			
*int*1	*int*1 F	GCCTTGATGTTACCCGAGAG	196	Barraud et al.[8]	22.5
	*int*1 R	GATCGGTCGAATGCGTGT			

Relative gene abundances were calculated for selected samples (Figures S3, S4) and expressed as gene copies per bacterial cell. The number of bacterial cells was estimated based on the absolute abundances of the 16S rRNA genes and the average number of 16S rRNA copies per bacterial genome of 4.1[9, 10].

1.4. ASSESSMENT OF MICROBIAL VIABILITY

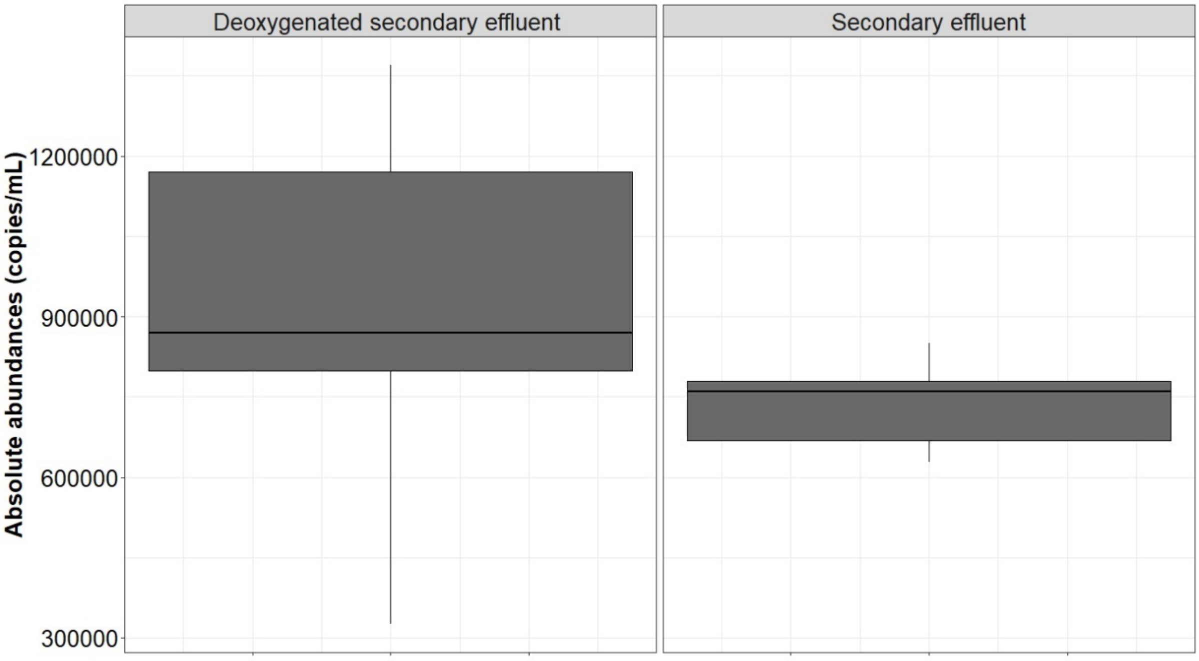

Figure S1. Triplicate samples of secondary clarifier effluent (feedwater) were either deoxygenated (left) and stored aerobically (right) and then treated with propidium monoazide (PMA) for determining the number of viable bacteria. Absolute concentrations of the 16S rRNA gene (copies/mL) in both samples were statistically not significantly different ($p = 0.2$), indicating that cell viability was not impacted by deoxygenation.

1.5. STATISTICAL ANALYSIS

All qPCR data were statistically tested with a significance cutoff of $\alpha = 0.05$. The Bartlett and Sapiro-Wilk tests were used to validate the assumption that sample populations tested were homogeneous and normally distributed, respectively. If both assumptions were met, the data were assumed homogeneous and normally distributed, and significant differences were assessed using one-way analysis of variance (ANOVA) and Tukey post-hoc test for pairwise comparisons. Non-parametric tests, such as Krustall-Wallis and Games-Howell post-hoc tests, were performed to determine significant differences and assess pairwise comparisons, respectively, when the assumptions for normality and homoscedasticity were violated in either log or square root transformed data.

2. RESULTS

2.1. BENCHMARKING ARG GENE REMOVAL BY H_2O_2/NAU-1 AGAINST CONVENTIONAL AOPS

Table S3. Mean removal (log_{10} values) of the target genes 16S rRNA, *int*1 and *tet*M monitored in benchmarking experiments with conventional AOPs UV and H_2O_2/UV against the novel mineral-based AOP H_2O_2/NAu-1. Standard deviations from the mean of three replicate experiments are provided. Conditions: 20 mM H_2O_2, 1 g/L NAu-1, 8 h contact time.

AOP conditions	16S rRNA	*int*1	*tet*M
96 mJ/cm^2 UV	0.69 ± 0.08	0.07 ± 0.19	0.58 ± 0.08
288 mJ/cm^2 UV	1.56 ± 0.50	0.52 ± 0.47	1.03 ± 0.52
576 mJ/cm^2 UV	1.52 ± 0.05	0.45 ± 0.05	1.13 ± 0.12
H_2O_2/UV 96 mJ/cm^2	0.89 ± 0.29	0.99 ± 0.04	0.79 ± 0.10
H_2O_2/UV 288 mJ/cm^2	0.88 ± 0.05	1.10 ± 0.12	0.92 ± 0.12
H_2O_2/UV 576 mJ/cm^2	1.05 ± 0.33	1.08 ± 0.30	0.84 ± 0.10
H_2O_2/NAu-1	0.46 ± 0.13	1.27 ± 0.19	1.31 ± 0.21
NAu-1	-0.19 ± 0.34	0.17 ± 0.06	0.36 ± 0.22

Table S4. Pairwise comparison of means (Games-Howell post hoc test) of removal of target genes 16S rRNA, *int*1 and *tet*M in the benchmarking experiments for treatment with UV, H_2O_2/UV and H_2O_2/NAu-1. Conditions: 20 mM H_2O_2, 1 g/L NAu-1, 8 h contact time. Statistical significance is noted by p-values (sigificance levels: $p \leq 0.05$: yellow, $p \leq 0.01$: green).

**16S rRNA**	96 mJ/cm^2 UV	288 mJ/cm^2 UV	576 mJ/cm^2 UV	H_2O_2/96 mJ/cm^2 UV	H_2O_2/288 mJ/cm^2 UV	H_2O_2/576 mJ/cm^2 UV	H_2O_2/NAu-1	NAu--1
96 mJ/cm^2 UV		0.06	**<0.01**	0.62			0.09	
288 mJ/cm^2 UV			0.99		0.16		**0.02**	
576 mJ/cm^2 UV						0.13	**<0.01**	
H_2O_2/UV 96 mJ/cm^2					1	0.96	**0.02**	
H_2O_2/UV 288 mJ/cm^2						0.89	**<0.01**	
H_2O_2/UV 576 mJ/cm^2							0.06	
H_2O_2/NAu-1								**<0.01**
NAu-1								
***int*1**								
96 mJ/cm^2 UV		0.49	0.14	**0.01**			**<0.01**	
288 mJ/cm^2 UV			0.99		0.22		0.09	
576 mJ/cm^2 UV						**<0.0 1**	**<0.01**	
H_2O_2/UV 96 mJ/cm^2					0.41	0.97	**0.02**	
H_2O_2/UV 288 mJ/cm^2						1	0.41	
H_2O_2/UV 576 mJ/cm^2							0.76	
H_2O_2/NAu-1								**<0.01**
NAu-1								
***tet*M**								
96 mJ/cm^2 UV		0.29	**<0.01**	**0.04**			**<0.01**	
288 mJ/cm^2 UV			0.99		0.99		0.80	
576 mJ/cm^2 UV						0.07	0.61	
H_2O_2/UV 96 mJ/cm^2					0.40	0.95	**<0.01**	
H_2O_2/UV 288 mJ/cm^2						0.90	**0.01**	
H_2O_2/UV 576 mJ/cm^2							**<0.01**	
H_2O_2/NAu-1								**<0.01**
NAu-1								

2.2. ACTIVATION OF H_2O_2 USING NAU-1: EFFECT OF TREATMENT PARAMETERS

Table S5. Absolute abundances of target genes 16S rRNA, *int*1, *bla*$_{OXA-10}$, *tet*M and *tet*Q in H_2O_2/NAu-1 treatment experiments to assess the effect of H_2O_2 dose (0.1, 0.26, 10 mM). Standard deviations from the mean of three replicate experiments are provided. 'Feedwater' refers to secondary clarifier effluent and 'no treatment' to secondary clarifier effluent stirred at room temperature (22±2 °C) with no addition of H_2O_2 or NAu-1. Conditions: 0.5 g/L NAu-1, 8 h contact time.

AOP condition	16S rRNA 10^5 copies/mL	*int*1 10^3 copies/mL	*bla*$_{OXA-10}$ 10^1 copies/mL	*tet*M 10^2 copies/mL	*tet*Q 10^1 copies/mL
**Controls**					
Feedwater	157 ± 31	143± 38	84.4 ± 10.7	194 ± 24	104 ± 36
No treatment	157 ± 70	167 ± 62	255 ± 146	168 ± 42	145 ± 57
NAu-1	123 ± 34	148 ± 80	209 ± 789	106 ± 31	28.0 ± 7.5
**H_2O_2 alone**					
0.1 mM H_2O_2	167 ± 75	136 ± 129	116 ± 77	119 ± 39	54.4 ± 41.7
0.26 mM H_2O_2	94.9 ± 62.2	37.5 ± 4.9	81.4 ± 9.3	93.2 ± 43.2	51.4 ± 7.1
10 mM H_2O_2	34.3 ± 14.0	22.9 ± 15.5	37.6 ± 22.0	64.9 ± 21.4	35.0 ± 24.6
**H_2O_2/NAu-1**					
0.1 mM H_2O_2/NAu-1	221 ± 20	304 ± 376	191 ± 173	131 ± 49	45.8 ± 12.0
0.26 mM H_2O_2/NAu-1	22.4 ± 14.3	2.41 ± 0.89	16.2 ± 11.8	180 ± 139	9.45 ± 8.27
10 mM H_2O_2/NAu-1	7.50 ± 4.76	0.78 ± 0.36	5.53 ± 3.01	8.36 ± 4.88	2.59 ± 1.15

Table S6. Absolute abundances of target genes 16S rRNA, *int*1, *bla*$_{OXA-10}$, *tet*M and *tet*Q in H_2O_2/NAu-1 treatment experiments to assess the effect of contact time (30 min, 4 h, 24 h). Standard deviations from the mean of three replicate experiments are provided. 'Feedwater' refers to secondary clarifier effluent and 'no treatment' to secondary clarifier effluent stirred at room temperature (22±2 °C) with no addition of H_2O_2 or NAu-1. Conditions: 0.26 mM H_2O_2, 0.5 g/L NAu-1.

AOP condition	16S rRNA 10^7 copies/mL	*int*1 10^4 copies/mL	*bla*$_{OXA-10}$ 10^2 copies/mL	*tet*M 10^3 copies/mL	*tet*Q 10^2 copies/mL
**Controls**					
Feedwater	3.90 ± 1.69	12.8 ± 1.7	1.84 ± 0. 90	14.4 ± 7.2	7.18 ± 2.28
No treatment 24 h	28.7 ± 4.8	59.4 ± 3.7	10.1 ± 1.8	28.8 ± 6.2	23.0 ± 4.9
NAu-1 24 h	5.67 ± 2.55	19.2 ± 9.6	1.94 ± 1.11	4.13 ± 0.52	1.84 ± 0.81
**H_2O_2 alone**					
30 min	9.83 ± 2.88	23.7 ± 5.2	4.49 ± 1.02	25.2 ± 6.5	16.2 ± 4.1
4 h	19.4 ± 2.88	37.0 ± 3.6	7.60 ± 1.50	34.4 ± 4.7	25.7 ± 3.7
24 h	21.6 ± 5.37	18.7 ± 1.8	5.74 ± 0.69	28.3 ± 12.0	38.6 ± 13.3
**H_2O_2/NAu-1**					
30 min	2.20 ± 1.46	4.62 ± 3.84	0.74 ± 0.53	3.70 ± 2.59	2.67 ± 2.23
4 h	1.11 ± 1.01	1.57 ± 2.03	0.30 ± 0.31	1.60 ± 2.12	1.21 ± 1.09
24 h	4.28 ± 1.21	4.76 ± 0.84	1.20 ± 0.36	7.63 ± 2.97	4.73 ± 1.45

Table S7. Pairwise comparison of means (Games-Howell post hoc test) of absolute abdundances of the 16S rRNA, *int*1, *bla*$_{OXA-10}$, *tet*M and *tet*Q genes in the H_2O_2/NAu-1 experiments with varying H_2O_2 dose. Conditions: 0.5 g/L NAu-1, 8 h contact time. Statistical significance is noted by p-values (sigificance levels: $p \leq 0.05$: yellow, $p \leq 0.01$: green).

**16S rRNA**	Feedwater	No treatment	NAu-1	0.1 mM H_2O_2	0.26 mM H_2O_2	10 mM H_2O_2	0.1 mM H_2O_2/NAu-1	0.26 mM H_2O_2/NAu-1	10 mM H_2O_2/NAu-1
Feedwater		1	0.55	0.99	0.28	**<0.01**	0.98	**<0.01**	**<0.01**
No treatment			0.90	0.99	0.56	**0.01**	0.98	**<0.01**	**<0.01**
NAu-1							0.84	**<0.01**	**<0.01**
0.1 mM H_2O_2							0.99		
0.26 mM H_2O_2								**0.05**	
10 mM H_2O_2									**0.02**
0.1 mM H_2O_2/NAu-1								**0.01**	**0.01**
0.26 mM H_2O_2/NAu-1									0.18
***int*1**									
Feedwater		0.98	1	1	**0.01**	**<0.01**	0.91	**<0.01**	**<0.01**
No treatment			0.99	0.99	**<0.01**	**<0.01**	0.96	**<0.01**	**<0.01**
NAu-1							0.93	**<0.01**	**<0.01**
0.1 mM H_2O_2							0.91		
0.26 mM H_2O_2								**<0.01**	
10 mM H_2O_2									**0.03**
0.1 mM H_2O_2/NAu-1								**0.03**	**0.03**
0.26 mM H_2O_2/NAu-1									**<0.01**
***bla*$_{OXA-10}$**									
Feedwater		0.10	**0.02**	0.93	0.99	**<0.01**	0.65	**<0.01**	**<0.01**
No treatment			0.99	0.30	0.09	**0.02**	0.99	**0.01**	**0.01**
NAu-1							0.99	**<0.01**	**<0.01**
0.1 mM H_2O_2							0.94		
0.26 mM H_2O_2								**<0.01**	
10 mM H_2O_2									**0.03**
0.1 mM H_2O_2/NAu-1								**0.01**	**0.01**
0.26 mM H_2O_2/NAu-1									0.29
***tet*M**									
Feedwater		0.81	**<0.01**	0.09	**<0.01**	**<0.01**	0.09	**<0.01**	**<0.01**
No treatment			0.06	0.27	**0.03**	**<0.01**	0.74	**<0.01**	**<0.01**
NAu-1							0.90	**<0.01**	**<0.01**
0.1 mM H_2O_2							0.99		
0.26 mM H_2O_2								**0.01**	
10 mM H_2O_2									**<0.01**
0.1 mM H_2O_2/NAu-1								**<0.01**	**<0.01**
0.26 mM H_2O_2/NAu-1									0.62
***tet*Q**									
Feedwater		0.73	**0.03**	0.33	0.13	**0.04**	0.09	**0.01**	**0.01**
No treatment			**<0.01**	**0.03**	**0.01**	**<0.01**	**0.01**	**<0.01**	**<0.01**
NAu-1							0.06	**<0.01**	**<0.01**
0.1 mM H_2O_2							0.99		
0.26 mM H_2O_2								**<0.01**	
10 mM H_2O_2									0.06
0.1 mM H_2O_2/NAu-1								**<0.01**	**<0.01**
0.26 mM H_2O_2/NAu-1									0.35

Table S8. Pairwise comparison of means (Games-Howell post hoc test) of absolute abdundances of the 16S rRNA, *int*1, *bla*$_{OXA-10}$, *tet*M and *tet*Q genes in the H_2O_2/NAu-1 experiments with varying contact time. Conditions: 0.5 g/L NAu-1, 0.26 mM H_2O_2. Statistical significance is noted by p-values (sigificance levels: $p \leq 0.05$: yellow, $p \leq 0.01$: green).

	Feedwater	No treatment 24 h	NAu-1 24 h	30 min H_2O_2	4 h H_2O_2	24 h H_2O_2	30 min H_2O_2/NAu-1	4 h H_2O_2/NAu-1	24 h H_2O_2/NAu-1
**16S rRNA**									
Feedwater		**<0.01**	0.77	**<0.01**	**<0.01**	**<0.01**	0.56	0.09	0.99
No treatment 24 h			**<0.01**						**<0.01**
NAu-1 24 h									0.84
30 min H_2O_2							**<0.01**		
4 h H_2O_2								**<0.01**	
24 h H_2O_2									**<0.01**
30 min H_2O_2/NAu-1								0.66	0.08
4 h H_2O_2/NAu-1									**<0.01**
***int*1**									
Feedwater		**<0.01**	0.60	**<0.01**	**<0.01**	**<0.01**	**<0.01**	**<0.01**	**<0.01**
No treatment 24 h			**<0.01**						**<0.01**
NAu-1 24 h									**0.02**
30 min H_2O_2							**<0.01**		
4 h H_2O_2								**<0.01**	
24 h H_2O_2									**<0.01**
30 min H_2O_2/NAu-1								0.50	1
4 h H_2O_2/NAu-1									**0.02**
***bla*$_{OXA-10}$**									
Feedwater		**<0.01**	0.99	**<0.01**	**<0.01**	**<0.01**	0.28	**0.04**	0.74
No treatment 24 h			**<0.01**						**<0.01**
NAu-1 24 h									0.61
30 min H_2O_2							**<0.01**		
4 h H_2O_2								**<0.01**	
24 h H_2O_2									**<0.01**
30 min H_2O_2/NAu-1								0.46	0.49
4 h H_2O_2/NAu-1									**<0.01**
***tet*M**									
Feedwater		**0.04**	0.15	0.18	**<0.01**	0.20	**0.01**	**0.04**	0.49
No treatment 24 h			**<0.01**						**<0.01**
NAu-1 24 h									0.10
30 min H_2O_2							**<0.01**		
4 h H_2O_2								**<0.01**	
24 h H_2O_2									**0.01**
30 min H_2O_2/NAu-1								0.63	0.13
4 h H_2O_2/NAu-1									**<0.01**
***tet*Q**									
Feedwater		**<0.01**	**0.01**	**<0.01**	**<0.01**	**<0.01**	**0.05**	**0.01**	0.41
No treatment 24 h			**<0.01**						**<0.01**
NAu-1 24 h									**<0.01**
30 min H_2O_2							**<0.01**		
4 h H_2O_2								**<0.01**	
24 h H_2O_2									**<0.01**
30 min H_2O_2/NAu-1								0.70	0.39
4 h H_2O_2/NAu-1									**<0.01**

Table S9. Removal (log values, see eq. 1 in main manuscript) of target genes 16S rRNA, *int*1, *bla*$_{OXA-10}$, *tet*M and *tet*Q in H_2O_2/NAu-1 treatment experiments. 'No treatment' refers to secondary clarifier effluent stirred at room temperature (22±2 °C) with no addition of H_2O_2 or NAu-1. Standard deviations from the mean of three replicate experiments are provided.

AOP condition	16S rRNA	*int*1	*bla*$_{OXA-10}$	*tet*M	*tet*Q
**Controls**					
No treatment, 8h	0.05 ± 0.25	-0.04 ± 0.16	-0.41 ± 0.27	0.08 ± 0.12	-0.12 ± 0.17
No treatment, 24 h	-0.86 ± 0.07	-0.67 ± 0.03	-0.73 ± 0.08	-0.29 ±0.10	-0.50 ± 0.09
NAu-1, 8 h	0.13 ± 0.14	0.04 ± 0.22	-0.37 ± 0.16	0.28 ± 0.13	0.59 ± 0.14
NAu-1, 24 h	-0.13 ± 0.19	-0.13 ± 0.22	0.05 ± 0.28	0.55 ± 0.05	0.54 ± 0.07
**H_2O_2 alone**					
dose: 0.26 mM H_2O_2					
30 min	-0.38 ± 0.13	-0.26 ± 0.10	-0.38 ± 0.09	-0.23 ± 0.12	-0.34 ± 0.11
4 h	-0.69 ± 0.06	-0.46 ± 0.04	-0.61 ± 0.08	-0.37 ± 0.06	-0.55 ± 0.06
24 h	-0.73 ± 0.09	-0.16 ± 0.04	-0.49 ± 0.05	-0.25 ±0.24	-0.70 ± 0.19
contact time: 8 h					
0.1 mM H_2O_2	0.02 ± 0.23	0.40 ± 0.75	0.05 ± 0.52	0.23 ± 0.15	0.50 ± 0.54
0.26 mM H_2O_2	0.33 ± 0.36	0.59 ± 0.06	0.02 ± 0.05	0.37 ± 0.22	0.31 ± 0.06
10 mM H_2O_2	0.71 ± 0.25	0.95 ± 0.45	0.44 ± 0.33	0.49 ± 0.11	0.84 ± 0.93
**H_2O_2/NAu-1**					
dose: 0.26 mM H_2O_2					
30 min	0.40 ± 0.45	0.96 ± 1.09	0.59 ± 0.52	0.76 ± 0.47	0.65 ± 0.52
4 h	0.71 ± 0.40	1.65 ± 1.12	1.07 ± 0.56	1.49 ± 0.78	0.94 ± 0.41
24 h	-0.02 ± 0.14	0.44 ± 0.08	0.21 ± 0.15	0.32 ± 0.21	0.20 ± 0.13
contact time: 8 h					
0.1 mM H_2O_2/NAu-1	-0.02 ± 0.34	0.03 ± 0.59	-0.21 ± 0.37	0.20 ± 0.16	0.37 ± 0.11
0.26 mM H_2O_2/NAu-1	0.93 ± 0.30	1.80 ± 0.17	0.81 ± 0.29	1.18 ± 0.40	1.18 ± 0.36
10 mM H_2O_2/NAu-1	1.43 ± 0.34	2.34 ± 0.32	1.23 ± 0.21	1.43 ± 0.25	1.65 ± 0.22

Table S10. Pairwise comparison of means (Games-Howell post hoc test) of log removal of the 16S rRNA, *int*1, *bla*$_{OXA-10}$, *tet*M and *tet*Q genes in the H_2O_2/NAu-1 experiments with varying H_2O_2 dose. Conditions: 0.5 g/L NAu-1, 8 h contact time. Statistical significance is noted by p-values (sigificance levels: $p \leq 0.05$: yellow, $p \leq 0.01$: green).

**16S rRNA**	NAu-1	0.1 mM H_2O_2	0.26 mM H_2O_2	10 mM H_2O_2	0.1 mM H_2O_2/NAu-1	0.26 mM H_2O_2/NAu-1	10 mM H_2O_2/NAu-1
NAu-1					0.92	**<0.01**	**<0.01**
0.1 mM H_2O_2			0.42	**<0.01**	0.99	**<0.01**	**<0.01**
0.26 mM H_2O_2				0.25	0.46	**0.02**	**<0.01**
10 mM H_2O_2					**<0.01**	0.73	**<0.01**
0.1 mM H_2O_2/NAu-1						**<0.01**	**<0.01**
0.26 mM H_2O_2/NAu-1							0.07
***int*1**							
NAu-1					1	**<0.01**	**<0.01**
0.1 mM H_2O_2			0.99	0.57	0.93	**<0.01**	**<0.01**
0.26 mM H_2O_2				0.34	0.21	**<0.01**	**<0.01**
10 mM H_2O_2					**0.03**	**<0.01**	**<0.01**
0.1 mM H_2O_2/NAu-1						**<0.01**	**<0.01**
0.26 mM H_2O_2/NAu-1							**0.01**
***bla*$_{OXA-10}$**							
NAu-1					0.91	**<0.01**	**<0.01**
0.1 mM H_2O_2			0.99	0.57	0.91	**0.03**	**<0.01**
0.26 mM H_2O_2				**0.05**	0.64	**<0.01**	**<0.01**
10 mM H_2O_2					**0.02**	0.24	**<0.01**
0.1 mM H_2O_2/NAu-1						**<0.01**	**<0.01**
0.26 mM H_2O_2/NAu-1							**0.05**
***tet*M**							
NAu-1					0.90	**<0.01**	**<0.01**
0.1 mM H_2O_2			0.80	**0.01**	0.99	**<0.01**	**<0.01**
0.26 mM H_2O_2				0.79	0.58	**<0.01**	**<0.01**
10 mM H_2O_2					**<0.01**	**<0.01**	**<0.01**
0.1 mM H_2O_2/NAu-1						**<0.01**	**<0.01**
0.26 mM H_2O_2/NAu-1							0.79
***tet*Q**							
NAu-1					**0.02**	**<0.01**	**<0.01**
0.1 mM H_2O_2			0.95	0.97	0.99	0.09	**<0.01**
0.26 mM H_2O_2				0.68	0.81	**<0.01**	**<0.01**
10 mM H_2O_2					0.78	0.96	0.29
0.1 mM H_2O_2/NAu-1						**<0.01**	**<0.01**
0.26 mM H_2O_2/NAu-1							0.07

Table S11. Pairwise comparison of means (Games-Howell post hoc test) of log removal of the 16S rRNA, *int*1, *bla*$_{OXA-10}$, *tet*M and *tet*Q genes in the H_2O_2/NAu-1 experiments with varying contact time. Conditions: 0.5 g/L NAu-1, 0.26 mM H_2O_2. Statistical significance is noted by p-values (sigificance levels: $p \leq 0.05$: yellow, $p \leq 0.01$: green).

**16S rRNA**	NAu-1 8 h	NAu-1 24 h	30 min H_2O_2/NAu-1	4 h H_2O_2/NAu-1	8 h H_2O_2/NAu-1	24 h H_2O_2/NAu-1
NAu-1 8 h					**<0.01**	
NAu-1 24 h						0.93
30 min H_2O_2/NAu-1				0.86	0.17	0.27
4 h H_2O_2/NAu-1					0.92	**<0.01**
8 h H_2O_2/NAu-1						**<0.01**
***int*1**						
NAu-1 8 h					**<0.01**	
NAu-1 24 h						0.85
30 min H_2O_2/NAu-1				0.69	0.97	0.55
4 h H_2O_2/NAu-1					0.95	**0.02**
8 h H_2O_2/NAu-1						**<0.01**
***bla*$_{OXA-10}$**						
NAu-1 8 h					**<0.01**	
NAu-1 24 h						**<0.01**
30 min H_2O_2/NAu-1				0.93	0.47	0.88
4 h H_2O_2/NAu-1					0.99	0.15
8 h H_2O_2/NAu-1						**<0.01**
***tet*M**						
NAu-1 8 h					**<0.01**	
NAu-1 24 h						0.15
30 min H_2O_2/NAu-1				0.38	0.59	0.32
4 h H_2O_2/NAu-1					0.97	**0.03**
8 h H_2O_2/NAu-1						**<0.01**
***tet*Q**						
NAu-1 8 h					**0.01**	
NAu-1 24 h						**<0.01**
30 min H_2O_2/NAu-1				0.93	0.32	0.36
4 h H_2O_2/NAu-1					0.92	**<0.01**
8 h H_2O_2/NAu-1						**<0.01**

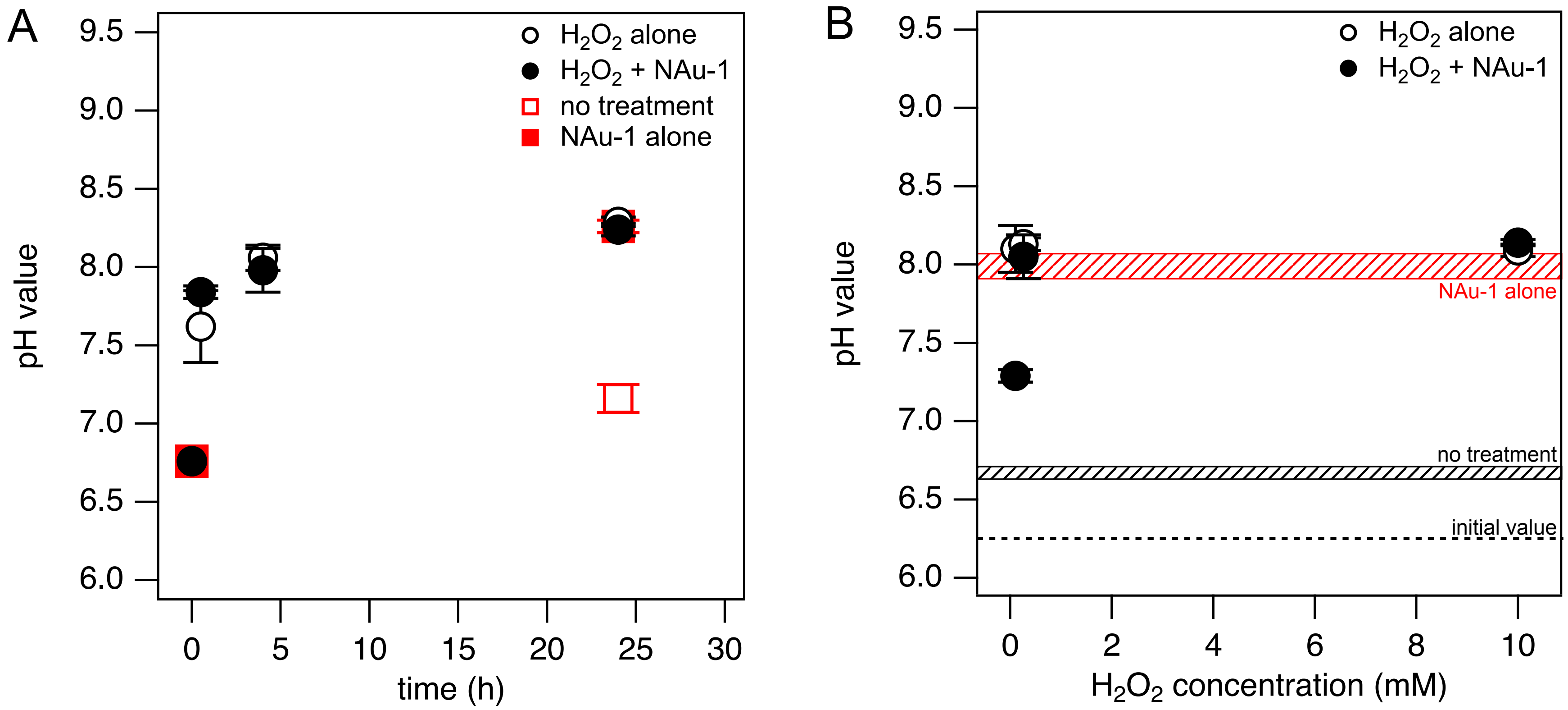

Figure S2. Measured pH values in secondary clarifier effluent during treatment with H_2O_2/NAu-1 in the absence (open markers; H_2O_2 alone) and presence of 0.5 g/L NAu-1 (filled markers; H_2O_2 + NAu-1), monitored for (A) different contact times with a constant initial H_2O_2 dose of 0.26 mM and (B) as a function of initial H_2O_2 dose after 8 h contact time. Values for the no treatment control and NAu-1 only addition are added in (A) as markers and in (B) as ranges (value ± standard deviation). Error bars represent standard deviations from the mean of three replicate experiments.

Table S12. Aqueous Fe concentrations in secondary clarifier effluent (feedwater) during treatment with H_2O_2/NAu-1. Standard deviations from the mean of three replicate experiments are provided. Only one sample of feedwater was analysed.

AOP condition	Fe(II) µM	Fe(total) µM
**Contact time: 8 h**		
Controls		
Feedwater (t = 0)	16.1	40.7
NAu-1	0.73 ± 0.00	1.06 ± 0.14
Treatment		
0.1 mM H_2O_2/NAu-1	0.65 ± 0.14	0.98 ± 0.24
0.26 mM H_2O_2/NAu-1	0.89 ± 0.14	1.06 ± 0.14
10 mM H_2O_2/NAu-1	0.49 ± 0.00	0.85 ± 0.17
**dose: 0.26 mM H_2O_2**		
Controls		
Feedwater (t = 0)	1.38	2.28
NAu-1, 24 h	0.75 ± 0.13	1.39 ± 0.00
Treatment		
30 min	1.00 ± 0.13	1.86 ± 0.07
4 h	0.92 ± 0.07	1.60 ± 0.07
24 h	0.71 ± 0.14	1.56 ± 0.41

Table S13. Pairwise comparison of means (Games-Howell post hoc test) of log removal amongst the target genes 16S rRNA, *int*1, *bla*$_{OXA-10}$, *tet*M and *tet*Q in the H_2O_2/NAu-1 experiments with varying contact time (conditions: 0.5 g/L NAu-1, 0.26 mM H_2O_2.) and varying H_2O_2 dose (conditions: 0.5 g/L NAu-1, 8 h contact time). Statistical significance is noted by p-values (sigificance levels: $p \leq 0.05$: yellow, $p \leq 0.01$: green).

**30 min**	16S rRNA	*int*1	*bla*$_{OXA-10}$	*tet*M	*tet*Q
16S rRNA		0.97	0.92	0.49	0.81
*int*1			0.99	0.78	0.97
bla$_{OXA-10}$				0.95	0.99
*tet*M					0.98
*tet*Q					
**4 h**					
16S rRNA		0.87	0.54	0.11	0.75
*int*1			0.89	0.25	0.99
bla$_{OXA-10}$				0.68	0.97
*tet*M					0.36
*tet*Q					
**8 h**					
16S rRNA		**0.01**	0.90	0.59	0.50
*int*1			**<0.01**	0.16	0.15
bla$_{OXA-10}$				0.22	0.16
*tet*M					0.99
*tet*Q					
**24 h**					
16S rRNA		**<0.01**	**0.02**	**<0.01**	**0.01**
*int*1			0.12	0.72	0.08
bla$_{OXA-10}$				0.72	0.99
*tet*M					0.60
*tet*Q					
**0.1 mM H_2O_2**					
16S rRNA		0.95	0.79	0.48	0.06
*int*1			0.69	1	0.96
bla$_{OXA-10}$				0.07	**0.01**
*tet*M					0.1
*tet*Q					
**0.26 mM H_2O_2**					
16S rRNA		**0.01**	0.91	0.59	0.51
*int*1			**<0.01**	0.17	0.15
bla$_{OXA-10}$				0.23	0.17
*tet*M					1
*tet*Q					
**10 mM H_2O_2**					
16S rRNA		0.15	0.61	0.45	0.49
*int*1			**0.05**	**0.03**	0.4
bla$_{OXA-10}$				0.8	**0.01**
*tet*M					0.13
*tet*Q					

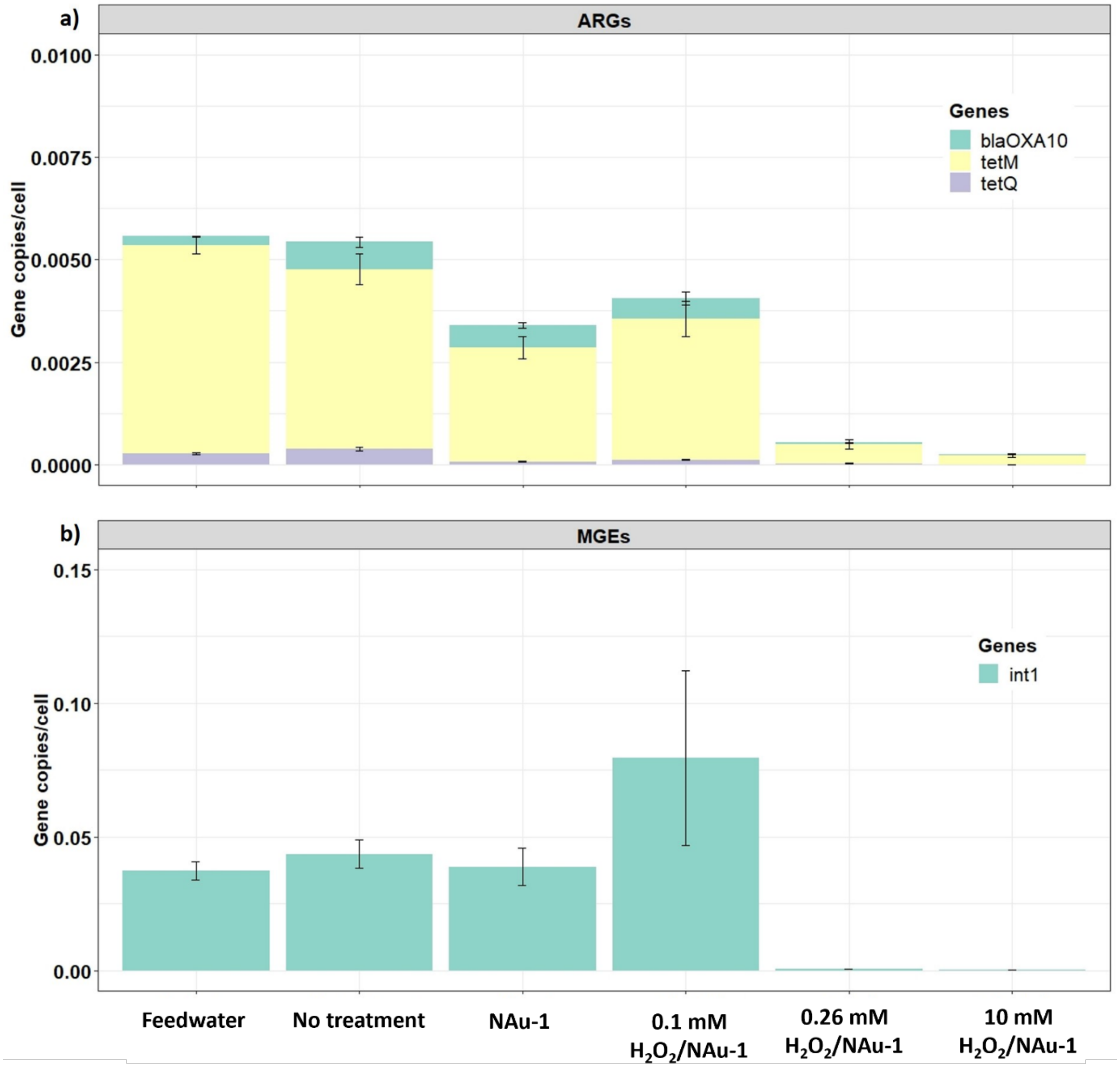

Figure S3. Relative abundances per bacterial cell (see Section 1.3) of a) the ARGs *bla*$_{OXA-10}$, *tet*M and *tet*Q, and b) the MGE *int*1 in the H_2O_2/NAu-1 experiments with varying H_2O_2 dose. Error bars represent standard deviations from the mean of three replicate experiments. 'Feedwater' refers to secondary clarifier effluent and 'no treatment' to secondary clarifier effluent stirred at room temperature (22±2 °C) with no addition of H_2O_2 or NAu-1. Conditions: 0.5 g/L NAu-1, 8 h contact time.

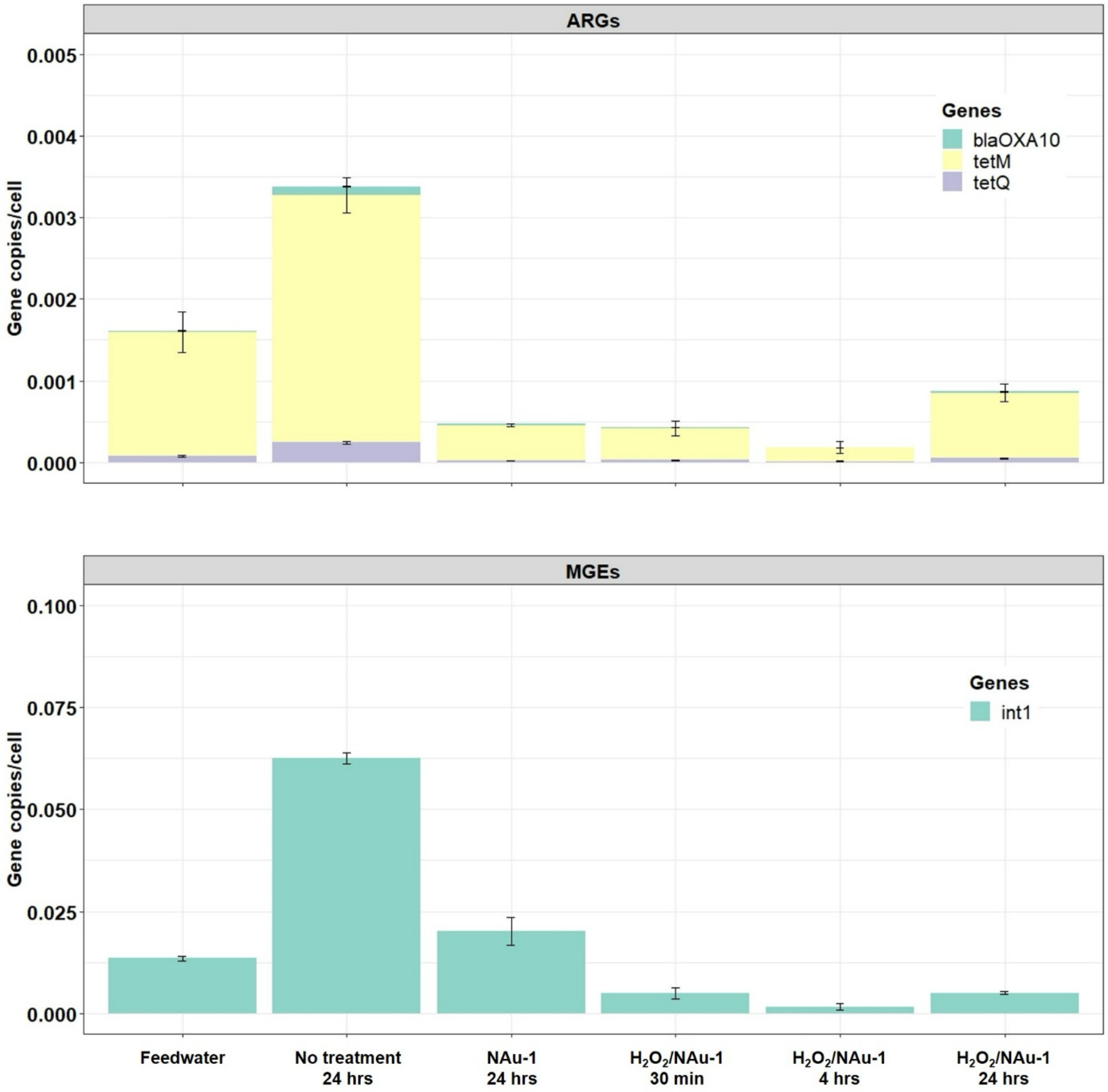

Figure S4. Relative abundances per bacterial cell (see Section 1.3) of a) the ARGs *bla*$_{OXA-10}$, *tet*M and *tet*Q, and b) the MGE *int*1 in the H_2O_2/NAu-1 experiments with contact time. Error bars represent standard deviations from the mean of three replicate experiments. ‘Feedwater’ refers to secondary clarifier effluent and ‘no treatment’ to secondary clarifier effluent stirred at room temperature (22±2 °C) with no addition of H_2O_2 or NAu-1. Conditions: 0.5 g/L NAu-1, 0.26 mM H_2O_2.

Table S14. Wastewater parameters pH, chemical oxygen demand (COD), UV absorbance (at 254 nm), total phosphorous (TP), total dissolved solids (TSS) and total organic carbon (TOC) during H_2O_2/NAu-1 treatment under pseudo-optimal conditions (0.26 mM H_2O_2, 0.5 g/L NAu-1, 4-8 h contact time). Standard deviations from the mean of three replicate experiments are provided.

Contact time<br>Parameter	0 h	4 h	8 h
pH	6.9 ± 0.1	7.6 ± 0.2	7.9 ± 0.1
COD (mg/L)	30.5 ± 0.2	28.7 ± 0.7	28.4 ± 3.5
UV absorbance at 254 nm (a.u.)	0.100 ± 0.004	0.040 ± 0.021	0.040 ± 0.011
TSS (mg/L)	7.50 ± 2.04	6.25 ± 3.75	22.1 ± 6.5
TOC (mg/L)	10.3 ± 0.05	12.1 ± 0.8	13.2 ± 0.3
TP (mg/L)	1.73 ± 0.00	1.29 ± 0.04	1.27 ± 0.04

2.3. TREATMENT USING REDUCED NAU-1

Table S15. Absolute abundances of target genes 16S rRNA, *int*1, *bla*$_{OXA-10}$, *tet*M and *tet*Q in treatment experiments with 0.5 g/L rNAu-1 under oxic (ambient air) and anoxic conditions (<2 ppm O_2). Standard deviations from the mean of three replicate experiments are provided. 'Feedwater' refers to deoxygenated secondary clarifier effluent and 'no treatment' to secondary clarifier effluent in the absence of rNAu-1 stirred at room temperature (22±2 °C) for the indicated time.

AOP condition	16S rRNA	*int*1	*bla*$_{OXA-10}$	*tet*M	*tet*Q
	10^4 copies/mL	10^4 copies/mL	10^1 copies/mL	10^2 copies/mL	10^2 copies/mL
**Controls**					
Effluent	207 ± 25	86.6± 58.0	92.3 ± 6.9	686 ± 235	142 ± 56
Feedwater	314 ± 95	97.2 ± 30.3	114 ± 28	567 ± 293	201 ± 65
**Oxic**					
No treatment controls					
8 h	491 ± 109	157 ± 27	149 ± 23	932 ± 170	187 ± 28
24 h	259 ± 55	99.9 ± 15.9	105 ± 21	443 ± 100	48.2 ± 8.0
rNAu-1					
30 min	27.6 ± 39.8	15.7 ± 23.0	8.49 ± 11.8	34.0 ± 43.4	10.8 ± 15.3
4 h	0.69 ± 0.17	0.28 ± 0.08	0.86 ± 0.28	2.92 ± 1.54	0.47 ± 0.22
8 h	92.2 ± 137	40.4 ± 60.0	35.4 ± 47.4	94.4 ± 14.9	26.7 ± 39.1
24 h	1.31 ± 0.36	0.47 ± 0.19	1.28 ± 0.56	3.35 ± 1.31	0.26 ± 0.15
**Anoxic**					
No treatment controls					
8 h	372 ± 147	86.2 ± 18.5	123 ± 36	629 ± 179	136 ± 31
24 h	351 ± 51	128 ± 23	158 ± 21	442 ± 40	138 ± 18
rNAu-1					
8 h	6.49 ± 8.95	0.43 ± 0.26	1.67 ± 1.94	3.75 ± 4.20	1.74 ± 1.76
24 h	20.1 ± 29.0	7.80 ± 11.4	10.7 ± 14.1	16.7 ± 24.3	4.83 ± 6.64

Table S16. Pairwise comparison of means (Games-Howell post hoc test) of absolute abundances of the 16S rRNA, int1, blaOXA-10, tetM and tetQ genes in treatment experiments with 0.5 g/L rNAu-1 under oxic (ambient air) and anoxic conditions (<2 ppm O2). 'Feedwater' refers to deoxygenated secondary clarifier effluent and 'no treatment' to secondary clarifier effluent in the absence of rNAu-1. Statistical significance is noted by p-values (significance levels: p ≤ 0.05: yellow, p ≤ 0.01: green).

			**Oxic**						**Anoxic**			
	Effluent	Feedwater	No treatment 8 h	No treatment 24 h	30 min	4 h	8 h	24 h	No treatment 8 h	No treatment 24 h	8 h	24 h
**16S rRNA**												
Effluent		0.17										
Feedwater			0.06	0.91	**<0.01**	**<0.01**	**0.03**	**<0.01**	0.99	0.99	**<0.01**	**<0.01**
**Oxic** No treatment 8 h							**<0.01**					
No treatment 24 h								**<0.01**				
30 min						0.67	0.94	0.69				
4 h							0.68	**0.01**				
8 h								0.69			0.75	
24 h												0.71
**Anoxic** No treatment 8 h											**<0.01**	
No treatment 24 h												**<0.01**
8h												
***int*1**												
Effluent		0.99										
Feedwater			**0.01**	1	**<0.01**	**<0.01**	0.40	**<0.01**	0.99	0.45	**<0.01**	**<0.01**
**Oxic** No treatment 8 h							**<0.01**					
No treatment 24 h								**<0.01**				
30 min						0.67	0.98	0.69				
4 h							0.68	0.30				
8 h								0.68			0.68	
24 h												0.71
**Anoxic** No treatment 8 h											**<0.01**	
No treatment 24 h												**<0.01**
8h												
***bla*OXA-10**												
Effluent		0.55										
Feedwater			0.23	0.99	**<0.01**	**<0.01**	**0.04**	**<0.01**	0.99	**0.05**	**<0.01**	**<0.01**
**Oxic** No treatment 8 h							**<0.01**					
No treatment 24 h								**<0.01**				
30 min						0.72	0.88	0.77				
4 h							0.65	0.67				
8 h								0.66			0.68	
24 h												0.68
**Anoxic** No treatment 8 h											**<0.01**	
No treatment 24 h												**<0.01**
8h												

		Effluent	Feedwater	Oxic: No treatment 8 h	Oxic: No treatment 24 h	Oxic: 30 min	Oxic: 4 h	Oxic: 8 h	Oxic: 24 h	Anoxic: No treatment 8 h	Anoxic: No treatment 24 h	Anoxic: 8 h	Anoxic: 24 h
***tet*M**													
	Effluent		0.99										
	Feedwater			0.20	0.98	**0.02**	**0.01**	**0.05**	**0.02**	0.99	0.97	**0.02**	**0.02**
**Oxic**	No treatment 8 h							**<0.01**					
	No treatment 24 h								**<0.01**				
	30 min						0.61	0.98	0.62				
	4 h							0.76	0.99				
	8 h								0.77			0.77	
	24 h												0.85
**Anoxic**	No treatment 8 h											**<0.01**	
	No treatment 24 h												**<0.01**
	8h												
***tet*Q**													
	Effluent		0.99										
	Feedwater			0.20	0.98	**0.02**	**0.01**	**0.05**	**0.02**	0.99	0.97	**0.02**	**0.02**
**Oxic**	No treatment 8 h							**<0.01**					
	No treatment 24 h								**<0.01**				
	30 min						0.61	0.98	0.62				
	4 h							0.76	0.99				
	8 h								0.77			0.77	
	24 h												0.85
**Anoxic**	No treatment 8 h											**<0.01**	
	No treatment 24 h												**<0.01**
	8h												

Table S17. Removal (log values, see eq. 1 in main manuscript) of target genes 16S rRNA, *int*1, bla_{OXA-10}, *tet*M and *tet*Q in rNAu-1 treatment experiments. 'No treatment' refers to deoxygenated secondary clarifier effluent stirred at room temperature (22±2 °C) with no addition of rNAu-1. Standard deviations from the mean of three replicate experiments are provided.

AOP condition	16S rRNA	*int*1	bla_{OXA-10}	*tet*M	*tet*Q
**Oxic**					
No treatment controls					
8 h	-0.04 ± 0.17	0.06 ± 0.09	-0.02 ± 0.12	-0.03 ± 0.12	0.18 ± 0.11
24 h	-0.05 ± 0.06	-0.11 ± 0.08	-0.14 ± 0.06	0.11 ± 0.04	0.17 ± 0.06
rNAu-1					
30 min	1.91 ± 1.02	1.73 ± 1.07	1.72 ± 0.79	1.61 ± 0.62	1.98 ± 0.88
4 h	2.67 ± 0.10	2.57 ± 0.12	2.15 ± 0.16	2.35 ± 0.25	2.67 ± 0.20
8 h	1.79 ± 1.34	1.59 ± 1.29	1.31 ± 1.01	1.82 ± 1.17	1.81 ± 1.06
24 h	2.39 ± 0.11	2.36 ± 0.20	1.98 ± 0.17	2.26 ± 0.19	2.94 ± 0.24
**Anoxic**					
No treatment controls					
8 h	-0.18 ± 0.10	-0.20 ± 0.08	-0.11 ± 0.07	-0.21 ± 0.08	0.03 ± 0.06
24 h	0.09 ± 0.09	-0.01 ± 0.07	0.04 ± 0.09	0.12 ± 0.10	0.62 ± 0.07
rNAu-1					
8 h	2.26 ± 0.78	2.41 ± 0.22	2.15 ± 0.56	2.46 ± 0.53	2.28 ± 0.46
24 h	2.06 ± 1.04	2.04 ± 1.10	1.53 ± 0.73	2.51 ± 1.16	2.22 ± 0.81

Table S18. Pairwise comparison of means (Games-Howell post hoc test) of log removal of the 16S rRNA, *int*1, bla_{OXA-10}, *tet*M and *tet*Q genes in treatment experiments with 0.5 g/L rNAu-1 under oxic (ambient air) and anoxic conditions (<2 ppm O_2). Statistical significance is noted by p-values (sigificance levels: $p \leq 0.05$: yellow, $p \leq 0.01$: green).

	Oxic				*Anoxic*	
	30 min	4 h	8 h	24 h	8 h	24 h
**16S rRNA**						
oxic						
30 min		0.51	1	0.90	0.99	0.99
4 h			0.64	0.06	0.83	0.74
8 h				0.90	0.99	0.99
24 h					0.99	0.98
Anoxic						
8 h						0.99
***int*1**						
oxic						
30 min		0.45	1	0.75	0.68	0.99
4 h			0.48	0.25	0.67	0.88
8 h				0.73	0.67	0.99
24 h					0.99	0.99
Anoxic						
8 h						0.98
**bla_{OXA-10}**						
oxic						
30 min		0.82	0.99	0.98	0.92	0.99
4 h			0.47	0.53	1	0.38
8 h				0.69	0.56	0.99
24 h					0.99	0.72
Anoxic						
8 h						0.59
***tet*M**						
Oxic						
30 min		0.11	0.99	0.18	0.12	0.58
4 h			0.92	0.99	0.99	0.99
8 h				0.96	0.87	0.95
24 h					0.98	0.99
Anoxic						
8 h						1
***tet*Q**						
Oxic						
30 min		0.46	0.99	0.16	0.99	0.99
4 h			0.42	0.29	0.40	0.81
8 h				0.17	0.95	0.99
24 h					0.06	0.35
Anoxic						
8 h						1

References

(1) Schaefer, M.V.;Gorski, C.A. Scherer, M.M. Spectroscopic evidence for interfacial Fe (II)− Fe (III) electron transfer in a clay mineral. *Environmental science & technology* **2011**, *45* (*2*)(540-545).

(2) Rancourt, D.G. Ping, J.Y. Voigt-based methods for arbitrary-shape static hyperfine parameter distributions in Mössbauer spectroscopy. *Nuclear Instruments and Methods in Physics Research Section B: Beam Interactions with Materials and Atoms* **1991**, *58* (*1*)(85-97).

(3) Rancourt, D. Lagarec, K., *Recoil, Mössbauer Spectral Analysis Software for Windows*. 1998, Version.

(4) Green, M.R. Sambrook, J., *Molecular cloning : a laboratory manual* 4th ed. 2012, N.Y.: Cold Spring Harbor Laboratory Press.

(5) Harms, G.;Layton, A.C.;Dionisi, H.M.;Gregory, I.R.;Garrett, V.M.;Hawkins, S.A.;Robinson, K.G. Sayler, G.S. Real-time PCR quantification of nitrifying bacteria in a municipal wastewater treatment plant. *Environmental Science and Technology* **2003**, *37* (*2*)(343-351).

(6) Wang, J.;Mao, D.;Mu, Q. Luo, Y. Fate and proliferation of typical antibiotic resistance genes in five full-scale pharmaceutical wastewater treatment plants. *Science of the Total Environment* **2015**, *526* (366-373).

(7) Peak, N.;Knapp, C.W.;Yang, R.K.;Hanfelt, M.M.;Smith, M.S.;Aga, D.S. Graham, D.W. Abundance of six tetracycline resistance genes in wastewater lagoons at cattle feedlots with different antibiotic use strategies. *Environmental Microbiology* **2007**, *9* (*1*)(143-151).

(8) Barraud, O.;Baclet, M.C.;Denis, F. Ploy, M.C. Quantitative multiplex real-time PCR for detecting class 1, 2 and 3 integrons. *Journal of Antimicrobial Chemotherapy* **2010**, *65* (*8*)(1642-1645).

(9) Klappenbach, J.A.;Saxman, P.R.;Cole, J.R. Schmidt, T.M. Rrndb: The ribosomal RNA operon copy number database. *Nucleic Acids Research* **2001**, *29* (*1*)(181-184).

(10) Quintela-Baluja, M.;Abouelnaga, M.;Romalde, J.;Su, J.-Q.;Yu, Y.;Gomez, M.L.;Smets, B.;Zhu, Y.-G. Graham, D.W. Spatial ecology of a wastewater network defines the antibiotic resistance genes in downstream receiving waters. *Water Res.* **2019**, *162* (347-357).
